# Growth performance and gut health of broilers fed graded inclusion levels of soyhulls in low-protein soybean meal diets and challenged with mixed *Eimeria* spp

**DOI:** 10.1016/j.psj.2026.107410

**Published:** 2026-07-08

**Authors:** Bhargavi Kasireddy, Iyabo W. Oluseyifunmi, Revathi Shanmugasundaram, Adelumola Oladeinde, Oluyinka A. Olukosi

**Affiliations:** a Department of Poultry Science, University of Georgia, Athens, GA 30602, USA; b Toxicology and Mycotoxin Research Unit, USDA-ARS, Athens, GA 30606, USA; c Egg and Poultry Production Safety Research Unit, USDA-ARS, Athens, GA 30606, USA

**Keywords:** *Eimeria*, Low-protein soybean meal, Soyhull, Growth performance, Inflammatory cytokines

## Abstract

The 42-d study investigated the effects of standard soybean meal (**SSBM**, 464 g/kg crude protein) or low-protein SBM (**LPSBM**) produced by adding soyhulls (**SH**) at 27, 55, or 86 g/kg (yielding 449, 437, and 417 g/kg crude protein, respectively; LPSBM 1-3) on growth performance and gut health in broilers challenged with mixed *Eimeria* spp. The aim was to investigate the impact of increasing dietary soybean fiber on growth, amino acid (**AA**) digestibility, and immune response in broiler chickens. 1,056 male broilers were allocated to 8 treatments in a 4 × 2 factorial arrangement, with 4 corn-based diets with or without a mixed-*Eimeria* challenge. All diets were balanced for ME, crude protein, and standardized digestible AA. The *Eimeria*-challenged birds were inoculated with a mixed-*Eimeria* spp. inoculum on d 14, and performance was recorded on 0, 10, 14, 17, 21, 28, and 42 d of age. During the acute phase (d 17-21), the challenge decreased body weight gain (BWG) and increased FCR (P ≤ 0.001). However, in the overall phase, *Eimeria*-challenged birds fed SSBM or LPSBM1 diets had higher BWG and final BW (*P* < 0.05). On d21, N, Tyr, and Met digestibility decreased linearly with the increasing dietary SH inclusion in the challenged birds (P < 0.05). The challenged birds had higher cecal isobutyrate levels and lower relative mRNA expression of jejunal PepT1, EAAT3, and OCLDN (P < 0.05). An interaction effect was observed for villi height: crypt depth, with non-challenged birds receiving LPSBM2 having a higher ratio than those fed the SSBM diet (P = 0.05). On d 21, challenged birds fed LPSBM1 had lower relative expression of cecal tonsils IL-1 and IFN-γ (P < 0.05). In conclusion, *Eimeria* challenge impaired growth performance. However, a moderate inclusion level of soybean fiber in birds fed the diet containing LPSBM1 enhanced growth performance metrics and positively modulated immune responses, whereas higher dietary SH levels (LPSBM2 and LPSBM3) negatively affected growth performance, particularly in the challenged birds.

## Introduction

Coccidiosis, caused by protozoa of the genus *Eimeria*, is one of the major threats to poultry health and productivity worldwide. Recent estimates indicate that the global chicken industry incurs about USD 14.5 billion in costs annually, mainly due to depressed growth rates and expenses associated with prevention and treatment (Blake et al., 2020). Coccidia infection damages intestinal epithelial cells, leading to osmotic imbalance, reduced nutrient absorption, and ultimately compromised performance in broiler chickens (Perez-Carbajal et al., 2010; Persia et al., 2006). Furthermore, the infection can deplete intracellular reservoirs of several bioactive nutrients and disrupt the gut microbiota equilibrium, predisposing birds to secondary infections by favoring the growth of opportunistic pathogens such as *Escherichia coli, Enterococcus, and Clostridium* (Campos et al., 2022). Growing global restrictions on the use of antibiotics in livestock production, such as poultry, drive the need for alternative strategies that support gut health and mitigate enteric diseases (Graham et al., 2023). Nutritional interventions, such as the inclusion of prebiotics, probiotics, enzymes, and functional fibrous ingredients, represent a promising strategy in this regard.

Agricultural by-products are emerging as important alternative feed ingredients in poultry nutrition, as the industry promotes increased sustainability and efficient utilization of feed resources. Although many by-products contain high levels of dietary fiber (**DF**), their moderate inclusion in the diet is often recommended due to their functional role in supporting gut health. A strategic use of high-fiber feedstuffs may therefore become increasingly important, as DF is increasingly recognized for its positive effects on overall bird health (Baurhoo et al., 2007; Craig et al., 2020). Studies have shown that moderate supplementation of fibrous components can support digestive tract development and function, thereby contributing to improved health and growth performance in chickens (Hetland and Svihus, 2001; Jiménez-Moreno et al., 2011; González-Alvarado et al., 2007). Both the DF type and the inclusion level can affect gastrointestinal tract development and growth performance in broiler chickens (Jiménez-Moreno et al., 2009).

Previous studies indicate that soluble fiber often contains water-holding compounds, mainly pectins, gums, and mucilages. These compounds can trap water and increase the digesta viscosity, which may slow its passage rate and affect how well nutrients are absorbed (Owusu-Asiedu et al., 2006; Perera et al., 2019). Conversely, insoluble fibers do not interfere with nutrient absorption, but they tend to accumulate in the gizzard, increasing the retention time and creating a reflux within the gastrointestinal tract, which may promote nutrient digestibility (Hetland et al., 2004; Mateos et al., 2012). A well-developed gizzard can also stimulate HCl secretion in the proventriculus, lower pH, and reduce pathogenic bacteria entering the digestive tract (Engberg et al., 2002). Furthermore, soluble fibers ferment more easily in the gut, whereas insoluble NSP are less fully hydrolyzed (Hetland et al., 2004; Montagne et al., 2003).

In this regard, soyhull (**SH**) is described as an insoluble low-lignified fiber source (Slama et al., 2019). Most previous studies have evaluated SH inclusions within diets containing conventional soybean meal (Ahsan et al., 2024; González-Alvarado et al., 2007; Tejeda and Kim, 2020). As defined in the current experiment, low-protein soybean meal (**LPSBM**) contains a larger percentage of SH, resulting in higher levels of insoluble fiber and lower crude protein (**CP**) content. Conventional or standard SBM, on the other hand, has comparatively lower fiber content because a significant quantity of the hulls is removed during processing. Insoluble fiber may have beneficial effects on gut health and nutrient utilization by improving gizzard function, regulating digesta passage rate, and helping maintain intestinal integrity during coccidial challenge. Consequently, we hypothesized that moderate inclusion of SH to generate LPSBM would improve growth performance, immune responses, and gut health in broiler chickens undergoing coccidial challenge, in a manner dependent on the SH inclusion level. Therefore, the objective of the study was to evaluate the effects of varying levels of SH inclusion in LPSBM on growth performance, nutrient digestibility, intestinal morphology, immune response, cecal microbial fermentation metabolite profile, and jejunal gene expression in broiler chickens challenged with coccidiosis.

## Materials and methods

The Institutional Animal Care and Use Committee of the University of Georgia approved all the experimental procedures used in this experiment (IACUC #: A2024 04-013)

### Birds, diets, and experimental design, and eimeria challenge

A total of 1056 male broiler chickens (by-product breeder chicks) were used in a 42-d floor pen study. Upon arrival on d 0, the birds were randomly allocated to eight experimental treatments, with six replicates per treatment in a randomized complete block design. Birds were fed in three phases: starter (d 0 to 10), grower (d 10 to 28), and finisher (d 28 to 42). Diet compositions for the starter are shown in Table 1, and for the grower and finisher phases are shown in Table 2. The starter phase diets were provided in crumble form, and the grower and finisher diets were provided as pellets. Three LPSBM (1-3) were produced by adding SH to standard soybean meal (**SSBM**) at inclusion levels of 27, 55, or 86 g/kg, yielding LPSBM1, 2, and 3, with CP contents of 449, 437, and 417 g/kg, respectively. The SSBM used in this study had an analyzed CP content of 464 g/kg. The intended target CP was 450, 440, and 430 g/kg for LPSBM1, 2, and 3, respectively. The treatments were arranged in a 4 × 2 factorial with diet type (SSBM, LPSBM1, LPSBM2, LPSBM3) and challenge status (non-challenged, **NCH**; or challenged, **CH**). The eight treatments were: 1) SSBM-NCH; 2) LPSBM1-NCH; 3) LPSBM2-NCH; 4) LPSBM3-NCH; 5) SSBM-CH; 6) LPSBM1-CH; 7) LPSBM2-CH; and 8) LPSBM3-CH. All diets were balanced for metabolizable energy, CP, and standardized digestible amino acids. Because the *Eimeria* challenge was administered on d 14, there were four dietary treatments (SSBM, LPSBM1, LPSBM2, and LPSBM3) during the pre-challenge phases. To calculate the ileal nutrient digestibility, titanium dioxide was added at 3 g/kg as a digestibility marker in the grower diets. All diets were supplemented with phytase (Quantum Blue, AB Vista) at 500 FTU/kg, with adjustments for available P and total Ca to match the phytase matrix values in the formulation. The temperature and lighting schedule during the experiment adhered to the Cobb 500 recommendations (Cobb, 2023).

**Table 1:** Feedstuff and chemical composition (g/kg, as fed) of the starter phase (d 0-10) diets.

Items	SSBM^1^	LPSBM1^1^	LPSBM2^1^	LPSBM3^1^
Corn	572.71	559.45	544.68	529.72
SSBM	365.00			
LPSBM1		375.00		
LPSBM2			385.00	
LPSBM3				395.00
Soybean oil	26.00	31.00	36.00	41.00
Dicalcium Phosphate	17.50	17.85	17.90	18.10
Limestone	5.90	5.80	5.80	5.70
L-Lys-HCl	2.00	1.10	0.90	0.80
DL-Met	1.60	1.60	1.62	1.58
L-Thr	1.89	0.80	0.70	0.70
Titanium dioxide	0.00	0.00	0.00	0.00
Sodium bicarbonate	2.00	2.00	2.00	2.00
Salt	2.80	2.80	2.80	2.80
Vitamin premix^2^	1.00	1.00	1.00	1.00
Trace minerals premix^3^	0.80	0.80	0.80	0.80
Choline chloride	0.70	0.70	0.70	0.70
Phytase (Quantum blue-5000G)^4^	0.10	0.10	0.10	0.10
Total	1000	1000	1000	1000
Calculated nutrients and energy, g/kg				
Protein	220	220	220	219
ME, kcal/kg	2906	2902	2898	2893
Ca	7.97	7.96	7.96	7.95
Available P	4.33	4.34	4.33	4.34
Acid detergent fiber	22.3	26.2	30.2	34.5
Neutral detergent fiber	58.1	62.7	67.4	72.4
Digestible amino acids, g/kg				
Arg	13.27	13.59	13.84	13.79
His	5.28	5.59	5.67	5.68
Ile	8.67	9.42	9.53	9.51
Leu	17.08	18.04	18.24	18.10
Lys	12.61	12.63	12.64	12.61
Met	4.82	4.79	4.81	4.80
Cys	2.70	3.26	3.30	3.33
Phe	9.88	10.42	10.59	10.50
Tyr	7.01	7.57	7.59	7.55
Thr	8.61	8.60	8.62	8.64
Trp	2.67	2.66	2.61	2.66
Val	9.35	10.19	10.31	10.28
TSAA	7.51	8.06	8.11	8.13
Phe+Tyr	16.89	17.99	18.18	18.05
SH inclusion in the total diet	0.00	9.86	20.21	31.13

1 SSBM - standard protein soybean meal (464g/kg); LPSBM - low-protein soybean meal (LPSBM1, 449g/kg; LPSBM2,437g/kg; LPSBM3, 417g/kg). The LPSBM1, 2, and 3 were produced by mixing soyhull at the rates of 27, 55, or 86 g/kg, respectively, with the SSBM.

2 Vitamin premix: Supplemented per kg of diet: Vitamin A, 5480 IU; Vitamin D3, 2640 IU; Vitamin E, 11 IU; Vitamin B12, 13.2 µg; menadione sodium bisulphite, 4.35 mg; Riboflavin, 5.49 mg; d- pantothenic acid, 11 mg; thiamine mononitrate, 2.2 mg; niacin, 44 mg; Vitamin B6, 3.3 mg; folic acid, 900 µg; biotin, 55.2 µg.

3 Mineral premix: Iodine, 0.15 mg; manganese, 66.06 mg; copper, 5 mg; iron,45 mg; zinc, 44.5 mg; selenium, 0.3 mg.

4 Quantum blue phytase: Phytase (AB Vista, Malborough, UK) supplemental dose of 0.1 g/kg to supply 500 FTU/ kg of feed.

**Table 2:** Feedstuff and chemical composition (g/kg, as fed) of the grower (d 10-28) and finisher phase (d 28-42) diets.

Items	Grower phase				Finisher phase			
	SSBM^1^	LPSBM1^1^	LPSBM2^1^	LPSBM3^1^	SSBM^1^	LPSBM1^1^	LPSBM2^1^	LPSBM3^1^
Corn	635.62	622.19	606.48	591.49	658.64	647.05	632.35	616.46
SSBM	310.00				285.00			
LPSBM1		320.00				293.00		
LPSBM2			330.00				303.00	
LPSBM3				340.00				314.00
Soybean oil	22.00	27.00	33.00	38.00	30	35	40	45
Dicalcium phosphate	7.50	7.80	7.80	8.00	6.00	6.20	6.30	6.40
Limestone	8.72	8.65	8.70	8.60	8.40	8.40	8.30	8.30
L-Lys.HCl	2.38	1.52	1.30	1.23	1.84	1.10	0.90	0.75
DL-Met	1.70	1.72	1.72	1.70	1.52	1.55	1.55	1.49
L-Thr	1.68	0.72	0.60	0.58	1.20	0.30	0.20	0.20
Titanium dioxide	3.00	3.00	3.00	3.00	0.00	0.00	0.00	0.00
Sodium bicarbonate	2.00	2.00	2.00	2.00	2.00	2.00	2.00	2.00
Salt	2.80	2.80	2.80	2.80	2.80	2.80	2.80	2.80
Vitamin premix^2^	1.00	1.00	1.00	1.00	1.00	1.00	1.00	1.00
Trace minerals premix^3^	0.80	0.80	0.80	0.80	0.80	0.80	0.80	0.80
Choline chloride	0.70	0.70	0.70	0.70	0.70	0.70	0.70	0.70
Phytase (Quantum blue-5000 G)^4^	0.10	0.10	0.10	0.10	0.10	0.10	0.10	0.10
Total	1000	1000	1000	1000	1000	1000	1000	1000
Calculated nutrients and energy								
Protein, g/kg	200	200	200	200	190	190	190	191
ME, kcal/kg	2953	2951	2955	2953	3033	3035	3035	3033
Ca, g/kg	6.35	6.35	6.36	6.36	5.77	5.78	5.76	5.77
Available P, g/kg	2.51	2.52	2.50	2.52	2.21	2.21	2.21	2.21
Acid Detergent Fiber, g/kg	23.2	26.5	29.9	33.6	23.4	26.4	29.6	33.0
Neutral Detergent Fiber, g/kg	63.1	66.8	70.6	74.8	64.8	68.2	71.7	75.5
Digestible amino acids, g/kg								
Arg	11.8	12.1	12.4	12.4	11.2	11.4	11.7	11.7
His	4.83	5.10	5.18	5.20	4.61	4.85	4.93	4.97
Ile	7.80	8.46	8.57	8.58	7.38	7.97	8.09	8.13
Leu	16.0	16.9	17.0	16.9	15.5	16.2	16.4	16.3
Lys	11.6	11.6	11.6	11.6	10.6	10.6	10.6	10.6
Met	4.70	4.71	4.71	4.72	4.41	4.42	4.43	4.40
Cys	2.56	3.05	3.08	3.11	2.49	2.93	2.96	3.00
Phe	12.0	12.5	12.6	12.6	8.52	8.94	9.11	9.09
Tyr	6.40	6.89	6.92	6.91	6.11	6.54	6.58	6.59
Thr	7.82	7.80	7.80	7.81	7.06	7.01	7.03	7.08
Trp	2.36	2.36	2.32	2.37	2.21	2.20	2.18	2.23
Val	8.54	9.27	9.39	9.39	8.15	8.80	8.93	8.95
Phe+Tyr	15.4	16.3	16.5	16.5	14.6	15.5	15.7	15.7
Soyhull inclusion in the total diet, g/kg	0.00	8.42	17.4	26.8	0.00	7.71	15.9	24.7

1 SSBM - standard protein soybean meal (464g/kg); LPSBM - low-protein soybean meal (LPSBM1, 449g/kg; LPSBM2,437g/kg; LPSBM3, 417g/kg). The LPSBM1, 2, and 3 were produced by mixing soyhull at the rates of 27, 55, or 86 g/kg, respectively, with the SSBM.

2 Vitamin premix: Supplemented per kg of diet: Vitamin A, 5480 IU; Vitamin D3, 2640 IU; Vitamin E, 11 IU; Vitamin B12, 13.2 µg; menadione sodium bisulphite, 4.35 mg; Riboflavin, 5.49 mg; d- pantothenic acid, 11 mg; thiamine mononitrate, 2.2 mg; niacin, 44 mg; Vitamin B6, 3.3 mg; folic acid, 900 µg; biotin, 55.2 µg.

3 Mineral premix: Iodine, 0.15 mg; manganese, 66.06 mg; copper, 5 mg; iron,45 mg; zinc, 44.5 mg; selenium, 0.3 mg.

4 Quantum blue phytase: Phytase (AB Vista, Malborough, UK) supplemental dose of 0.1 g/kg to supply 500 FTU/ kg of feed.

On d 14, birds in the CH group were gavaged orally with 12,500 sporulated oocysts of *E. tenella*, 12,500 sporulated oocysts of *E. maxima*, and 62,500 sporulated oocysts of *E. acervulina* in 1 ml of distilled water to produce a mild infection. The birds in the NCH group were gavaged with 1 ml of distilled water.

### Growth performance and sample collection

Birds and feed were weighed on a pen basis on d 0, 10 (starter phase), 14 (pre-challenge), 17 (pre-patent), 21 (acute phase), 28 (recovery phase), and 42 (compensatory growth) to assess body weight gain (**BWG**), feed intake (**FI**), and FCR. Mortality was recorded daily and used to adjust growth performance parameters. Ileal digesta were collected from five birds per pen on d 14 and 21, pooled by pen for each sampling day, and freeze-dried for nutrient digestibility analysis. Samples for gene expression analyses were collected from the jejunum on d 21, and from the spleen and cecal tonsil on d 21 and 28. These samples were snap-frozen in liquid nitrogen and stored at −80°C until analysis.

Mid-jejunal tissue was collected for morphology measurements, including villus height (**VH**), crypt depth (**CD**), and the VH/CD ratio. The effects of treatments on immune responses on d 21 and 28 were also analyzed in the spleen and cecal tonsils for CD4+ and CD8+ cells, and in the bile for anti-coccidia immunoglobulins. Cecal contents were collected from one randomly selected bird per pen on d 21 for short-chain fatty acid (**SCFA**) analysis and stored at −20°C until further analysis. On d 42, two birds per pen were randomly selected, weighed, and euthanized to determine the relative weights of gizzard, spleen, and bursa of Fabricius.

### Chemical analysis

All chemical analyses (unless otherwise stated) were conducted according to the (AOAC 2016) methods. Freeze-dried ileal digesta samples, along with diet samples, were ground to 0.5 mm to analyze for DM, N, amino acids, and titanium dioxide. Dry matter in diet and digesta samples was determined by drying samples in a drying oven (Avantor, Radnor, PA, USA) at 100 °C for 24 h (Method 934.01). The concentration of titanium dioxide in the sample was analyzed as outlined by (Short et al., 1996). Determination of N content in diets and digesta was performed by the combustion method (Method 990.03), using a LECO analyzer (LECO Corporation, St Joseph, MI, USA), and the CP level was calculated using a factor of 6.25. Acid detergent fiber and neutral detergent fiber of diets were analyzed by Ankom 200 Fiber Analyzer (Ankom Technology, Macedon, NY, USA). Samples for amino acid analysis were hydrolyzed in 6 N hydrochloric acid (Method 982.30) for 24 h at 110°C under N. Performic acid oxidation (Method 994.12) was used for Met and Cys prior to acid hydrolysis. The quantification was done using high-performance liquid chromatography (HPLC) following post-column derivatization (Method 982.30E a, b, c).

### Short-chain fatty acids

Short-chain fatty acids were analyzed using procedures previously outlined (Lourenco et al., 2020). Approximately 1 g of cecal content sample was diluted with 3 ml of distilled water (1:3 ratio) and vortexed. A 1.5 ml of vortexed sample was centrifuged at 10,000 × g for 10 min. Then, 1 ml of supernatant was mixed with 0.2 ml of 25% (wt/v) metaphosphoric acid solution and frozen overnight. After thawing, the samples were centrifuged again at 10,000 × g for 10 min, and the supernatant was mixed with an internal standard in a 5:1 ratio. Ethyl acetate was used to dilute the mixture in a 1:2 ratio (1 part mixture to 2 parts ethyl acetate), vortexed, and allowed to settle for 5 min. The top layer of the mixture, about 900 µl, was collected and analyzed by gas chromatography (Shimadzu GC-2010 plus; Shimadzu Corporation, Kyoto, Japan) using a flame ionization detector and a capillary column (Zebron ZB-FFAP; 30 m × 0.32 mm × 0.25 μm; Phenomenex Inc., Torrance, CA, USA). Helium was used as the carrier gas, and the oven temperature was gradually increased from 110 to 200°C while maintaining the detector and injector temperatures at 350°C and 250°C, respectively. The SCFA levels were calculated by comparing sample peak heights with standard peak heights.

### Jejunal Histomorphology

On d 21, mid-jejunal samples approximately 2 cm in length were collected from one bird per pen, cut, and fixed in 10% neutral buffered formalin. The samples were processed using a tissue processor and embedded in paraffin as described earlier (Shanmugasundaram et al., 2013). Hematoxylin and eosin were used to stain paraffin blocks after they were sectioned at 5 µm and placed on Superfrost slides. The VH, CD, and VH/CD were measured using Keyence imaging software on three properly orientated cross-sections.

### Relative gene expression analysis

Relative mRNA expression analysis of target genes of jejunal tight junction proteins, nutrient transporters, and pro- and anti-inflammatory cytokine expression in spleen (systemic response) and cecal tonsil (local response) was performed using Quantitative real-time PCR. Tissue samples of approximately 60-80 mg were homogenized by QiAzol lysis reagent (QIAGEN, Hilden, Germany) using a bead beater with zirconia/silica beads, and the total RNA was extracted according to the manufacturer’s instructions. The RNA purity and concentration were assessed using a NanoDropTM Eight Spectrophotometer (Thermo Fisher Scientific, Wilmington, DE 19810, USA). RNA concentrations were determined from absorbance 260 nm and normalized to 2 µg/µl. A high-capacity cDNA reverse transcription kit (Thermo Fisher Scientific, Waltham, MA) was used to synthesize cDNA from extracted RNA in a 20 µl reaction volume. Quantitative reverse transcriptase polymerase chain reaction (qRT-PCR) was performed with SYBR Green Master mix (Bio-Rad, Hercules, CA) in the Step One Plus real-time PCR system. Samples were run in duplicates and analyzed by qRT-PCR using the 2^-ΔΔCt^ method (Livak and Schmittgen, 2001), using glyceraldehyde-3-phosphate dehydrogenase (GAPDH) as the housekeeping gene and SSBM-NCH as the reference treatment. The target primers, housekeeping genes, and their accession numbers are listed in Supplementary Table 1.

### Relative gizzard, spleen, and bursa of Fabricius weights

On d 42, two birds per pen were randomly selected, weighed, and euthanized by cervical dislocation. Gizzard contents were emptied and the weight of spleen, gizzard, and bursa of fabricius were expressed as relative weights according to the following equation:

$$Organ\;index=\;\frac{Organ\;weight,\;g}{Live\;body\;weight,\;kg}$$

### Spleen and cecal tonsil CD8+:CD4+ ratio

On d 21 and 28 (acute and recovery phases, respectively), we measured the effect of feeding LPSBM in broiler chickens on the percentages of CD4+ and CD8+ cells in the spleen and cecal tonsil from one randomly selected bird per pen using flow cytometry, as previously described (Shanmugasundaram et al., 2015). After preparing single-cell suspensions from the spleen and cecal tonsils, mononuclear cells were separated using density centrifugation over Histopaque (1.077 g/mL, Sigma-Aldrich, St. Louis, MO) for 15 min at 400 × g. Later, the cells were incubated (15 min) with a 1:250 dilution of fluorescent-isothiocyanate-conjugated mouse anti-chicken CD4+ (Southern Biotech, Birmingham, AL), a 1:200 dilution of unlabeled mouse IgG, and a 1:450 dilution of phycoerythrin-conjugated mouse anti-chicken CD8+ (Southern Biotech, Birmingham, AL). The cells were washed with wash buffer after removing unbound antibodies by centrifugation, and the percentages of CD4+ and CD8+ cells were analyzed using a flow cytometer (Guava EasyCyte, Millipore, MA), and the CD8+:CD4+ ratio was calculated.

### Total Bile IgA quantification by ELISA

On d 21 and 28, bile was collected from the gall bladder of one randomly selected bird per pen and stored at –20°C for subsequent analysis. Total IgA levels in the bile were quantified using an indirect ELISA with a chicken IgA ELISA kit, following the manufacturer’s instructions (Bethyl Laboratories Inc., Montgomery, TX, USA, Catalogue # E33-103) as described earlier (Dasireddy et al., 2025). The plates were incubated for 1 h at room temperature after 100 μl of a 1:1600 dilution of bile in SuperBlock (PBS) Blocking Buffer was added in duplicate. Following a wash step, 100 μl of a 1:100,000 dilution of horseradish peroxidase-labeled anti-chicken IgA (Novus Biologicals, Littleton, CO, USA) in SuperBlock (PBS) Blocking Buffer was added and incubated at room temperature for 1 h. Plates were washed with PBS-Tween 20. Later, 100 μl of 3,3,5,5-tetramethylbenzidine (TMB) solution (eBioscience, San Diego, CA, USA) was added to each well. A microplate ELISA reader was used to measure the optical density at 450 nm after the reaction was stopped with 100 μl of 1 N HCl for 10 minutes. The mean optical density was used to express IgA levels.

### Antimicrobial resistance gene analysis

The relative abundance of antimicrobial resistance genes (AMR) in the caecal digesta was quantified by quantitative real-time PCR. Approximately 0.2 g of caecal contents were used for genomic DNA extraction using the QIAamp Fast DNA StoolMini Kit (QIAGEN, Hilden, Germany) following the manufacturer’s instructions. A NanoDrop Eight Spectrophotometer was used to measure the concentration and purity of DNA (Thermo Fisher Scientific, Wilmington, DE, U.S.A.). Quantitative PCR was performed with SsoAdvanced^TM^ Universal SYBR Green Supermix (Bio-Rad, Hercules, CA, U.S.A.). A Bio-Rad CFX96 Real-Time PCR Detection System was used to run each reaction in duplicates (Bio-Rad Laboratories, Hercules, CA, U.S.A.). Sulphonamide resistance gene 2 (sul2), tetracycline resistance gene M (tetM), streptomycin resistance gene B (strB), and β-lactamase CTX-M type gene (blaCTX-M) are among the target AMR genes, and the housekeeping gene for normalization used was glyceraldehyde-3-phosphate dehydrogenase A (gapA). By creating standard curves using known quantities of genomic DNA for each target gene, relative gene copy numbers were measured. All qPCR runs included no-template controls to verify the absence of contamination. Data from duplicate wells were averaged for each sample, and mean values per pen were used. The relative abundance of each AMR gene (gene copies/sample) was normalized against the abundance of gapA (gene copies/sample). The list of primers for AMR genes is provided in the Supplementary Table 2.

### Calculations

Nutrient digestibility was calculated using the following equations:

$$\text{Dry}\;\text{matter}\;\text{digestibility}\;\left(\text{DMD}\right)=\left[1-\left(\begin{array}{c} \frac{\mathrm{Ti}\;\mathrm{in}\;\mathrm{diet}}{\mathrm{Ti}\;\mathrm{in}\;\mathrm{digesta}}\\ \; \end{array}\right)\right]\times 100$$

$$\text{Nutrient}\;\text{digestibility}\;\left(\%\right)=\left[1-\;\left(\begin{array}{c} \frac{Ti\;in\;diet}{Ti\;in\;digesta}\\ \; \end{array}\right)\times \;\left(\frac{Nutrient\;in\;digesta}{Nutrient\;in\;diet}\right)\;\right]\times \;100$$

### Statistical analysis

Data were analyzed as appropriate for a randomized complete block design using JMP Pro 18. Orthogonal polynomial contrasts were used to determine the linear and quadratic responses to increasing levels of SH in the diets. When both linear and quadratic responses are significant, only the quadratic response is discussed. When there are no significant interactions, only the main effect means are presented and discussed (the simple effects are presented in the Supplementary tables). In addition to the p-values for the main effect, the contrasts reported are *Eimeria* × Diets (Quadratic) and *Eimeria* × Diets (Linear). Because four SH levels are implied in the four diets, the p-value for the main effect of diet was partitioned to Diets (Linear) and Diets (Quadratic). Statistical significance was set at P ≤ 0.05, and trends were set at 0.05 < P ≤ 0.10.

## Results

The analyzed composition of grower diets is listed in Table 3, whereas the analyzed CP and digestible AA contents of SSBM, SH and LPSBM (1-3) are presented in Supplementary Table 3. All diets were formulated to have similar CP and standardized ileal-digestible AA profiles. The inclusion of SH to produce LPSBM incorporated into the diets increased the total SH in the final diet. The addition of SH to produce LPSBM also increased both ADF and NDF contents of the final diets by approximately 45% and 18%, respectively, in LPSBM3, the diet containing approximately 25 g/kg SH (the highest dietary SH level) in the total diet.

**Table 3:** Analyzed chemical composition (g/kg) of the grower diets (as fed basis).

Items	SSBM^1^	LPSBM1^1^	LPSBM2^1^	LPSBM3^1^
Dry matter	876	880	877	880
Crude protein	200	200	204	203
Indispensable amino acids				
Arg	12.9	12.4	13.3	13.2
His	5.5	5.4	5.5	5.6
Ile	8.8	8.6	8.9	9.5
Leu	17.5	17.3	17.6	18.0
Lys	12.8	11.9	12.6	12.6
Met	4.7	4.5	4.5	4.5
Phe	10.1	9.9	10.3	10.5
Thr	8.9	8.1	8.2	8.1
Trp	2.3	2.3	2.4	2.4
Val	9.5	9.4	9.6	10.2
Dispensable amino acids				
Ala	10.1	10.1	10.2	10.3
Asp	19.9	19.5	20.6	20.8
Cys	3.2	3.0	3.2	3.2
Glu	36.4	35.7	37.1	37.5
Gly	8.3	8.2	8.4	8.5
Pro	11.3	11.3	11.5	11.6
Ser	9.0	9.0	9.0	8.8
Tyr	7.1	7.0	7.3	6.9

1 Diets: SSBM – standard-protein soybean meal (464 g/kg); LPSBM - low-protein soybean meal (LPSBM1, 449 g/kg; LPSBM2, 437g/kg; LPSBM3, 417 g/kg). The LPSBM1, 2, and 3 were produced by mixing soyhull at the rates of 27, 55, or 86 g/kg, respectively, with the SSBM.

### Growth performance

Increasing SH inclusion levels across diets by using LPSBM significantly influenced BWG and FI during the starter and early-grower phases in the broiler chickens (Table 4). Both weight gain and FI decreased linearly from SSBM to LPSBM3 during the starter phase (P ≤ 0.001) and the early grower phase (P = 0.002), respectively. Birds fed the SSBM diet had higher BWG and FI during the early growth phases than those fed LPSBM2 and 3. However, there was no dietary effect on FCR during the starter and early grower phases.

**Table 4:** Growth performance response of broiler chickens fed diets containing standard or low-protein soybean meals during starter and early grower phases.

	Starter phase (d 0-10)				Early grower phase (d 10-14)			
Diets^1^	Weight Gain, g	Feed intake, g	FCR	Day 10 BW, g	Weight Gain, g	Feed Intake, g	FCR	Day 14 BW, g
SSBM	299	350	1.17	341	184	236	1.29	522
LPSBM1	284	338	1.19	325	179	230	1.29	500
LPSBM2	265	321	1.21	306	167	216	1.29	473
LPSBM3	267	318	1.19	309	165	214	1.30	471
Pooled SEM	5.39	5.38	0.016	5.46	4.70	4.64	0.017	9.51
	Probabilities							
Diet- Linear	< 0.001	< 0.001	0.263	< 0.001	0.002	0.002	0.489	0.001
Diet- Quadratic	0.124	0.351	0.333	0.124	0.768	0.672	0.769	0.309

1 Diets: SSBM- standard-protein soybean meal (464 g/kg); LPSBM - low-protein soybean meal (LPSBM1, 449 g/kg; LPSBM2,437 g/kg; LPSBM3, 417 g/kg). The LPSBM1, 2, and 3 were produced by mixing soyhull at the rates of 27, 55, or 86 g/kg, respectively, with the SSBM. The *Eimeria* challenge was done on d 14 with mixed species (12,500 sporulated oocysts of *E. maxima*, 12,500 sporulated oocysts of *E. tenella,* and 62,500 sporulated oocysts of *E. acervulina*) by oral gavage. n = 12 replicate pens per treatment, and each pen had 22 birds per replicate.

During the pre-patent phase (d 14 - 17), SH inclusion (SSBM to LPSBM3) linearly decreased (P < 0.01) BWG and FI and linearly increased FCR (Table 5). During the acute phase (d 17 - 21), birds in the CH group had lower BWG and FI and a higher FCR (P ≤ 0.001). Weight gain decreased linearly with dietary SH inclusion (SSBM to LPSBM3) during the recovery phase (d 21 – 28), and the birds in the CH group had lower BWG (P < 0.001) without any effect on FI.

**Table 5:** Growth performance response during pre-patent, acute, and recovery phases of infection of broiler chickens challenged or unchallenged with mixed *Eimeria* spp. and fed diets containing standard or low-protein soybean meals.

		Pre-patent phase (d14-17)				Acute phase (d17-21)				Recovery phase (d21-28)			
*Eimeria*	Diets^1^	WG, g	FI, g	FCR	BW, g	WG, g	FI, g	FCR	BW, g	WG, g	FI, g	FCR	BW, g
	Means for the main effect of *Eimeria* challenge												
Challenged		297	416	1.40	794	49.6	207	5.62	841	678	1038	1.54	1517
Non-challenged		298	412	1.38	784	293	401	1.38	1077	737	1149	1.56	1802
		Means for the main effect of Diets											
	SSBM	311	423	1.36	832	169	312	3.09	998	731	1093	1.49	1723
	LPSBM1	303	423	1.40	803	176	304	4.78	979	706	1039	1.47	1679
	LPSBM2	291	404	1.39	765	171	305	2.87	935	703	1197	1.70	1631
	LPSBM3	285	405	1.43	756	168	296	3.26	924	690	1046	1.53	1604
		*Pooled standard error of the means*											
*Eimeria* challenge		3.61	3.95	0.013	9.06	5.38	5.01	0.814	11.7	7.44	48.5	0.067	14.0
Diets		5.10	5.59	0.018	12.8	7.61	7.09	1.15	16.6	10.5	68.6	0.095	19.8
		*Probabilities*											
*Eimeria* challenge		0.814	0.486	0.222	0.482	<0.001	<0.001	0.001	<0.001	<0.001	0.116	0.879	<0.001
Diet- Linear		0.001	0.007	0.019	<0.001	0.817	0.123	0.785	0.001	0.009	0.930	0.399	<0.001
Diet- Quadratic		0.920	0.959	0.907	0.441	0.525	0.925	0.573	0.789	0.565	0.484	0.419	0.651

1 Diets: SSBM- standard-protein soybean meal (464 g/kg); LPSBM - low-protein soybean meal (LPSBM1, 449 g/kg; LPSBM2,437 g/kg; LPSBM3, 417 g/kg). The LPSBM1, 2, and 3 were produced by mixing soyhull at the rates of 27, 55, or 86 g/kg, respectively, with the SSBM; WG – body weight gain; FI – feed intake, BW – body weight at the end of the period. The *Eimeria* challenge was done on d 14 with mixed species (12,500 sporulated oocysts of *E. maxima*, 12,500 sporulated oocysts of *E. tenella,* and 62,500 sporulated oocysts of *E. acervulina*) by oral gavage. n = 12 replicate pens for the main effect of diets and 24 replicate pens for the main effect of *Eimeria* challenge. Each pen had 22 birds. Only the main effects means are presented in the main document. Simple effect means are presented in Supplementary Table 4.

During the compensatory growth phase (Table 6), the birds in the CH group had greater weight gain irrespective of diet type (P = 0.029). A significant interaction effect (P = 0.022) was observed for the overall (d 0 – 42) BWG and FCR, and d 42 body weight. The birds in the CH group fed LPSBM2 or LPSBM3 had lower BWG and lower d 42 body weight (P < 0.05), and higher FCR (P = 0.059), with no treatment differences in the NCH group.

**Table 6:** Growth performance response during the compensatory and overall growth phases for broiler chickens challenged or unchallenged with mixed *Eimeria* spp. and fed diets containing standard or low-protein soybean meals.

		Compensatory growth phase (d28-42)				Overall phase (d 0-42)			
*Eimeria*	Diets^1^	Weight Gain, g	Feed intake, g	FCR	d 42 BW, g	Weight Gain, g	Feed intake, g	FCR	Final BW
		Means for the main effect of *Eimeria* challenge							
Challenged		1903	2908	1.54	3419	3383	5134	1.52	3424
Non-challenged		1791	2873	1.61	3592	3565	5383	1.51	3606
		Means for the main effect of Diets							
	SSBM	1840	2888	1.58	3561	3532	5302	1.50	3574
	LPSBM1	1897	2935	1.56	3576	3542	5270	1.49	3584
	LPSBM2	1818	2909	1.61	3450	3415	5351	1.57	3457
	LPSBM3	1833	2830	1.56	3435	3406	5109	1.50	3447
		Means for the simple effects							
Challenged	SSBM	1913	2911	1.53	3504	3475	5105	1.47	3516
	LPSBM1	2041	2992	1.48	3592	3555	5197	1.47	3596
	LPSBM2	1841	2931	1.60	3312	3273	5255	1.61	3314
	LPSBM3	1817	2798	1.55	3268	3230	4977	1.54	3271
Non-challenged	SSBM	1767	2865	1.62	3618	3590	5500	1.53	3631
	LPSBM1	1754	2878	1.64	3559	3530	5343	1.51	3571
	LPSBM2	1796	2887	1.62	3588	3558	5448	1.53	3599
	LPSBM3	1848	2862	1.56	3602	3582	5242	1.47	3623
		*Pooled standard error of the means*							
*Eimeria*		34.6	27.2	0.030	33.3	33.7	57.0	0.021	33.7
Diet		48.9	38.5	0.043	47.1	47.7	80.6	0.030	47.7
Interaction		69.1	54.4	0.061	66.6	67.4	114	0.042	67.4
		*Probabilities*							
*Eimeria*		0.029	0.367	0.106	0.001	0.001	0.004	0.708	0.001
Diet- Linear		0.649	0.250	0.950	0.022	0.023	0.173	0.572	0.023
*Eimeria* × Diet- Linear		0.084	0.251	0.298	0.027	0.022	0.634	0.049	0.022

1 Diets: SSBM- standard-protein soybean meal (464 g/kg); LPSBM - low-protein soybean meal (LPSBM1, 449 g/kg; LPSBM2,437 g/kg; LPSBM3, 417 g/kg). The LPSBM1, 2, and 3 were produced by mixing soyhull at the rates of 27, 55, or 86 g/kg, respectively, with the SSBM. The *Eimeria* challenge was done on d 14 with mixed species (12,500 sporulated oocysts of *E. maxima*, 12,500 sporulated oocysts of *E. tenella,* and 62,500 sporulated oocysts of *E. acervulina*) by oral gavage. n = 6 replicate pens for the simple effects, 12 replicate pens for the main effect of diets, and 24 replicate pens for the main effect of *Eimeria* challenge. Each pen had 22 birds. Linear and quadratic contrasts were used for the main effects of Diets and the *Eimeria* × Diet interaction. The quadratic effects are not reported because they were not significant.

### Relative spleen, gizzard, and bursa of Fabricius weights

Relative organ weights were unaffected by diet × challenge interactions (Table 7). The *Eimeria* challenge significantly increased the relative gizzard weight (P = 0.003), regardless of diet type. There was no significant treatment effect on the relative spleen weight. However, there was a quadratic response of relative bursa weight to diet type, increasing quadratically from LPSBM1 to LPSBM3 (P = 0.019), irrespective of challenge.

**Table 7:** Relative organ weight and jejunal histomorphology response (d 21) of broiler chickens challenged or unchallenged with mixed *Eimeria* spp. and fed diets containing standard or low-protein soybean meals.

		Relative organ weight, g/kg BW			Jejunal morphology		
*Eimeria*	Diets^1^	Gizzard	Spleen	Bursa	VH, µm	CD, µm	VH:CD^2^
		Means for the main effect of *Eimeria* challenge					
Challenged		12.01	1.15	1.27	978	369	2.68
Non-challenged		10.92	1.13	1.23	1581	279	5.97
		Means for the main effect of Diets					
	SSBM	11.62	1.12	1.23	1160	331	3.61
	LPSBM1	11.17	1.21	1.18	1270	320	4.37
	LPSBM2	11.78	1.11	1.28	1394	318	5.05
	LPSBM3	11.28	1.14	1.32	1294	326	4.28
		Means for the simple effects					
Challenged	SSBM	12.32	1.12	1.11	960	348	2.81
	LPSBM1	11.53	1.20	1.30	968	370	2.65
	LPSBM2	11.96	1.17	1.38	1007	387	2.65
	LPSBM3	12.21	1.13	1.29	978	369	2.63
Non-challenged	SSBM	10.91	1.12	1.34	1360	314	4.41
	LPSBM1	10.80	1.22	1.05	1572	269	6.09
	LPSBM2	11.59	1.05	1.18	1782	248	7.45
	LPSBM3	10.36	1.15	1.35	1610	283	5.94
		*Pooled standard error of the means*					
*Eimeria*		0.243	0.046	0.054	59.1	14.7	0.250
Diet		0.343	0.065	0.077	83.6	20.8	0.353
Interaction		0.486	0.093	0.109	118	29.5	0.500
		*Probabilities*					
*Eimeria*		0.003	0.757	0.636	< 0.001	0.001	0.001
Diet- Quadratic		0.122	0.609	0.019	0.216	0.639	0.037
*Eimeria* × Diet- Linear		0.800	0.903	0.264	0.253	0.305	0.046
*Eimeria* × Diet- Quadratic		0.945	0.609	0.569	0.307	0.156	0.023

1 Diets: SSBM- standard-protein soybean meal (464 g/kg); LPSBM - low-protein soybean meal (LPSBM1, 449 g/kg; LPSBM2,437 g/kg; LPSBM3, 417 g/kg). The LPSBM1, 2, and 3 were produced by mixing soyhull at the rates of 27, 55, or 86 g/kg, respectively, with the SSBM. VH – villi height; CD – crypt depth; BW – body weight. Samples for organ index and jejunal histomorphology were collected on d 21. The *Eimeria* challenge was done on d 14 with mixed species (12,500 sporulated oocysts of *E. maxima*, 12,500 sporulated oocysts of *E. tenella,* and 62,500 sporulated oocysts of *E. acervulina*) by oral gavage. n = 6 replicate pens for the simple effects, 12 replicate pens for the main effect of diets, and 24 replicate pens for the main effect of *Eimeria* challenge. Each pen had 22 birds. Linear and quadratic contrasts were used for the main effects of Diets and the *Eimeria* × Diet interaction. The Diet-Linear effects are not reported because they were not significant.

### Jejunal tissue histomorphology

No significant interaction or dietary effects were observed on VH or CD (Table 7). The *Eimeria* challenge significantly decreased VH and increased CD (P ≤ 0.001). A significant interaction effect was observed for VH: CD, where birds in the NCH group receiving LPSBM2 had a higher ratio than birds receiving the SSBM diet, with no treatment effects in CH birds (P = 0.023).

### Nutrient digestibility

Dry matter and AA digestibility were not influenced by the diets on d 14 (Supplementary Table 5). There was significant Diet × *Eimeria* (P = 0.027) for N digestibility on d 21, with digestibility decreasing linearly with increasing SH levels in the CH birds (P = 0.027) but with no dietary effect in the NCH group (Table 8). No significant interaction or diet effects were observed for the digestibility of dispensable AA except Tyr, which decreased (P = 0.044) linearly in CH birds fed diets with increasing SH inclusion (LPSBM 1 to 3), with no such effects in NCH birds.

**Table 8:** Ileal dry matter, nitrogen, and dispensable amino acid digestibility (d 21) for broiler chickens challenged or unchallenged with mixed *Eimeria* spp. and fed diets containing standard or low-protein soybean meals.

*Eimeria*	Diets^1^	DMD	Nitrogen	Asp	Ser	Glu	Pro	Gly	Ala	Cys	Tyr
		Means for the main effect of *Eimeria* challenge									
Challenged		57.4	62.8	67.3	66.5	75.1	66.1	58.3	60.3	49.3	68.5
Non- challenged		57.3	72.9	74.5	72.1	81.6	71.4	66.2	73.0	53.1	76.0
		Means for the main effect of Diets									
	SSBM	59.4	70.7	72.9	71.6	79.9	70.5	64.9	68.9	54.0	74.1
	LPSBM1	57.4	66.0	70.3	68.7	77.7	68.0	61.7	66.0	49.5	72.2
	LPSBM2	59.5	71.2	71.9	70.3	79.3	70.2	63.8	67.9	53.6	73.6
	LPSBM3	53.0	63.7	68.4	66.5	76.5	66.5	58.6	64.0	47.8	69.0
		Means for the simple effects									
Challenged	SSBM	60.7	68.4	71.1	70.3	78.2	69.5	62.9	64.8	54.1	72.4
	LPSBM1	58.9	61.4	68.1	67.7	75.1	66.5	59.8	60.9	51.1	70.0
	LPSBM2	60.3	67.8	68.3	67.4	76.2	67.4	59.7	61.6	51.0	69.9
	LPSBM3	49.6	53.8	61.6	60.4	70.9	61.1	50.8	54.1	41.1	61.7
Non-challenged	SSBM	58.2	73.0	74.7	72.8	81.5	71.6	66.8	73.0	53.9	75.9
	LPSBM1	56.0	70.7	72.5	69.7	80.3	69.4	63.6	71.1	47.9	74.4
	LPSBM2	58.6	74.5	75.5	73.2	82.4	72.9	67.9	74.2	56.2	77.2
	LPSBM3	56.3	73.5	75.1	72.6	82.2	71.9	66.4	73.8	54.4	76.4
		*Pooled standard error of the means*									
*Eimeria*		1.47	1.46	1.34	1.35	1.10	1.32	1.66	1.60	1.95	1.38
Diet		2.08	2.06	1.90	1.90	1.56	1.87	2.34	2.26	2.75	1.96
Interaction		2.93	2.91	2.68	2.6	2.21	2.64	3.31	3.20	3.90	2.77
		*Probabilities*									
*Eimeria*		0.963	< 0.001	0.001	0.005	< 0.001	0.007	0.002	< 0.001	0.176	0.001
Diet- Linear		0.070	0.089	0.164	0.117	0.237	0.249	0.120	0.207	0.240	0.120
*Eimeria* × Diet- Linear		0.128	0.027	0.062	0.063	0.083	0.094	0.064	0.075	0.055	0.044

1 Diets: SSBM- standard-protein soybean meal (464 g/kg); LPSBM - low-protein soybean meal (LPSBM1, 449 g/kg; LPSBM2,437 g/kg; LPSBM3, 417 g/kg). The LPSBM1, 2, and 3 were produced by mixing soyhull at the rates of 27, 55, or 86 g/kg, respectively, with the SSBM. The *Eimeria* challenge was done on d 14 with mixed species (12,500 sporulated oocysts of *E. maxima*, 12,500 sporulated oocysts of *E. tenella,* and 62,500 sporulated oocysts of *E. acervulina*) by oral gavage. n = 6 replicate pens for the simple effects, 12 replicate pens for the main effect of diets, and 24 replicate pens for the main effect of *Eimeria* challenge. Each pen had 22 birds. Linear and quadratic contrasts were used for the main effects of Diets and the *Eimeria* × Diet interaction. The Quadratic effects are not reported because they were not significant. Data of ileal AA digestibility on d 14 (prior to *Eimeria* challenge) are presented in Supplementary Table 5.

There was significant Diet × *Eimeria* (P < 0.05) for Met, Lys, and Arg digestibility (Table 9). The digestibility decreased (P < 0.05) linearly with increasing dietary SH level for birds in the CH group, whereas no treatment effect was observed in the NCH birds. *Eimeria* challenge significantly reduced (P < 0.01) the digestibility of all dispensable and indispensable AA, except for Trp and Cys.

**Table 9:** Indispensable amino acid digestibility (d 21) for broiler chickens challenged or unchallenged with mixed *Eimeria* spp. and fed diets containing standard or low-protein soybean meals.

*Eimeria*	Diets^1^	Thr	Val	Met	Ile	Leu	Phe	Lys	His	Arg	Trp
		Means for the main effect of *Eimeria* challenge									
Challenged		58.0	61.6	74.8	64.1	66.5	67.9	68.1	67.4	78.5	78.5
Non- challenged		66.6	72.5	85.4	74.2	75.1	76.7	80.6	75.2	84.8	76.4
Diets		Means for the main effect of Diets									
	SSBM	64.5	69.3	81.9	71.5	72.8	74.2	76.6	72.9	83.2	78.5
	LPSBM1	61.5	66.2	79.8	68.2	69.9	71.5	74.2	70.7	81.5	77.4
	LPSBM2	63.9	68.4	80.9	70.2	71.8	73.3	75.3	72.5	82.5	78.4
	LPSBM3	59.2	64.4	77.8	66.7	68.5	70.2	71.2	68.9	79.4	75.5
		Means for the simple effects									
Challenged	SSBM	62.3	66.3	78.6	68.8	70.8	72.0	72.6	70.8	81.8	79.7
	LPSBM1	59.7	62.0	75.5	64.3	66.3	67.9	69.5	68.2	79.4	80.0
	LPSBM2	59.3	62.5	75.5	64.8	67.5	68.8	68.9	68.5	79.2	79.3
	LPSBM3	50.7	55.7	69.5	58.5	61.3	62.9	61.2	62.1	73.7	74.8
Non-challenged	SSBM	66.8	72.2	85.2	74.2	74.8	76.3	80.6	75.1	84.6	77.2
	LPSBM1	63.4	70.4	84.1	72.1	73.4	75.0	78.8	73.2	83.6	74.8
	LPSBM2	68.5	74.2	86.3	75.7	76.2	77.9	81.7	76.5	85.8	77.6
	LPSBM3	67.6	73.1	86.2	74.9	75.7	77.5	81.2	75.8	85.2	76.1
		*Pooled standard error of the means*									
*Eimeria*		1.69	1.57	1.21	1.51	1.46	1.37	1.51	1.31	1.08	1.08
Diet		2.39	2.22	1.71	2.14	2.06	1.94	2.13	1.85	1.53	1.52
Interaction		3.38	3.14	2.43	3.02	2.92	2.74	3.02	2.61	2.16	2.15
		*Probabilities*									
*Eimeria*		0.001	<0.001	<0.001	<0.001	<0.001	<0.001	<0.001	<0.001	<0.001	0.197
Diet- Linear		0.210	0.212	0.161	0.207	0.240	0.256	0.125	0.223	0.142	0.247
*Eimeria* × Diet- Linear		0.054	0.064	0.040	0.063	0.081	0.065	0.047	0.068	0.042	0.278

1 Diets: SSBM- standard-protein soybean meal (464 g/kg); LPSBM - low-protein soybean meal (LPSBM1, 449 g/kg; LPSBM2,437 g/kg; LPSBM3, 417 g/kg). The LPSBM1, 2, and 3 were produced by mixing soyhull at the rates of 27, 55, or 86 g/kg, respectively, with the SSBM. The *Eimeria* challenge was done on d 14 with mixed species (12,500 sporulated oocysts of *E. maxima*, 12,500 sporulated oocysts of *E. tenella,* and 62,500 sporulated oocysts of *E. acervulina*) by oral gavage. n = 6 replicate pens for the simple effects, 12 replicate pens for the main effect of diets, and 24 replicate pens for the main effect of *Eimeria* challenge. Each pen had 22 birds. Linear and quadratic contrasts were used for the main effects of Diets and the *Eimeria* × Diet interaction. The Quadratic effects are not reported because they were not significant. Data on ileal AA digestibility on d 14 (prior to Eimeria challenge) are presented in Supplementary Table 5.

### Cecal short-chain fatty acids

No significant interactions were observed for cecal SCFA on d 21 (Table 10). Birds in the CH group had higher (P < 0.01) levels of propionate, isobutyrate, isovalerate, and valerate, independent of dietary SH level.

**Table 10:** Short-chain fatty acid profile (d 21) of broiler chickens challenged or unchallenged with mixed *Eimeria* spp. and fed diets containing standard or low-protein soybean meals.

*Eimeria*	Diets^1^	Acetate	Propionate	Isobutyrate	Butyrate	Isovalerate	Valerate	Total SCFA
		Means for the main effect of *Eimeria* challenge						
Challenged		79.3	9.04	1.58	18.6	2.27	1.78	113
Non- challenged		77.2	3.91	0.70	18.4	0.72	1.04	102
		Means for the main effect of Diets						
	SSBM	83.3	6.48	1.03	22.0	1.34	1.44	116
	LPSBM1	78.2	6.59	1.30	16.3	1.60	1.47	106
	LPSBM2	75.5	6.27	1.11	19.1	1.57	1.33	105
	LPSBM3	75.9	6.56	1.12	16.5	1.45	1.39	103
		*Pooled standard error of the means*						
*Eimeria*		3.00	0.606	0.091	1.54	0.158	0.093	4.54
Diet		4.11	0.884	0.133	2.25	0.231	0.135	6.62
		*Probabilities*						
*Eimeria*		0.603	<0.001	<0.001	0.928	<0.001	<0.001	0.093
Diet- Linear		0.176	0.981	0.861	0.141	0.711	0.701	0.166

1 Diets: SSBM- standard-protein soybean meal (464 g/kg); LPSBM - low-protein soybean meal (LPSBM1, 449 g/kg; LPSBM2,437 g/kg; LPSBM3, 417 g/kg). The LPSBM1, 2, and 3 were produced by mixing soyhull at the rates of 27, 55, or 86 g/kg, respectively, with the SSBM. The *Eimeria* challenge was done on d 14 with mixed species (12,500 sporulated oocysts of *E. maxima*, 12,500 sporulated oocysts of *E. tenella,* and 62,500 sporulated oocysts of *E. acervulina*) by oral gavage. n = 12 replicate pens for the main effect of diets, and 24 replicate pens for the main effect of *Eimeria* challenge. Each pen had 22 birds. Linear and quadratic contrasts were used for the main effects of Diets and the *Eimeria* × Diet interaction. The Diet-Quadratic effects are not reported because they were not significant. Only the main effects means are presented in the table due to the lack of *Eimeria* × Diet interaction. For the simple effect means, see Supplementary Table 6.

### Jejunal tight junction proteins and nutrient transporters, and cecal abundance of antimicrobial-resistance genes

No significant interactions were observed for the relative expression of jejunal tight junction proteins and nutrient transporters, nor for the abundance of AMR genes in the ceca content (Table 11). The *Eimeria* challenge significantly reduced relative expression of jejunal PepT1, BO+AT, EAAT3, and OCLDN, but increased relative expression of GLUT1, irrespective of diet. *Eimeria* challenge significantly decreased blaCTXM (P = 0.002) and increased the abundance of sul2 (P = 0.029) and tetM (P = 0.037). A significant diet effect was also observed for sul2, with an increased abundance in birds fed LPSBM1, followed by a decrease as SH inclusion increased, independent of challenge (P = 0.047).

**Table 11:** Relative gene expression of jejunal tight junction proteins and nutrient transporters, and relative abundance of antimicrobial-resistant genes in the ceca of broiler chickens (d 21) challenged or unchallenged with mixed *Eimeria* spp. and fed diets containing standard or low-protein soybean meals.

Eimeria	Diets^1^	Nutrient transporter genes					Tight junction proteins	Anti-microbial resistant genes			
		PepT1^2^	CAT1^2^	BO+AT^2^	EAAT3^2^	GLUT1^2^	OCLDN^2^	sul2^2^	tetM^2^	blaCTXM^2^	strB^2^
		Means for the main effect of *Eimeria* challenge									
Challenged		0.50	0.64	0.42	0.51	2.42	0.50	0.84	−0.95	−0.47	0.99
Non- challenged		1.11	0.80	0.91	1.00	0.97	0.90	−0.07	−2.36	0.65	0.48
		Means for the main effect of Diets									
	SSBM	0.71	0.75	0.55	0.74	1.52	0.67	0.02	−1.42	0.17	0.61
	LPSBM1	0.75	0.76	0.63	0.69	1.70	0.65	0.97	−1.22	0.45	1.29
	LPSBM2	0.91	0.75	0.68	0.79	1.69	0.76	0.63	−2.05	0.11	0.49
	LPSBM3	0.86	0.63	0.78	0.80	1.87	0.72	−0.07	−1.95	−0.36	0.56
		*Pooled standard error of the means*									
*Eimeria*		0.086	0.063	0.076	0.085	0.219	0.054	0.283	0.464	0.243	0.238
Diet		0.128	0.093	0.113	0.128	0.332	0.079	0.400	0.655	0.344	0.337
		*Probabilities*									
*Eimeria*		<0.001	0.080	<0.001	<0.001	<0.001	<0.001	0.029	0.037	0.002	0.136
Diet- Quadratic		0.716	0.490	0.913	0.817	0.987	0.919	0.047	0.937	0.287	0.368

1 Diets: SSBM- standard-protein soybean meal (464 g/kg); LPSBM - low-protein soybean meal (LPSBM1, 449 g/kg; LPSBM2,437 g/kg; LPSBM3, 417 g/kg). The LPSBM1, 2, and 3 were produced by mixing soyhull at the rates of 27, 55, or 86 g/kg, respectively, with the SSBM.

2 Pep t1, Peptide transporter 1; CAT 1, Cationic amino acid transporter 1; BO+AT, Neutral amino acid transporter; EAAT3, Excitatory amino acid transporter; GLUT 1, Glucose transporter 1; OCLDN, Occludin; sul2- Sulphonamide resistance gene 2; tetM-Tetracycline resistance gene M; blaCTX-M- β-lactamase CTX-M type gene; strB -Streptomycin resistance gene B, gapA- glyceraldehyde-3-phosphate dehydrogenase. The *Eimeria* challenge was done on d 14 with mixed species (12,500 sporulated oocysts of *E. maxima*, 12,500 sporulated oocysts of *E. tenella,* and 62,500 sporulated oocysts of *E. acervulina*) by oral gavage. n = 12 replicate pens for the main effect of diets, and 24 replicate pens for the main effect of *Eimeria* challenge. Each pen had 22 birds. Linear and quadratic contrasts were used for the main effects of Diets and the *Eimeria* × Diet interaction. The Diet-Linear effects are not reported because they were not significant. Only the main effects means are presented in the table due to the lack of *Eimeria* × Diet interaction. For the simple effect means, see Supplementary Table 7.

### Pro- and anti-inflammatory cytokines expression in the spleen and cecal tonsils

On d 21, a significant interaction effect (P < 0.01) was observed for IL-1 and IFN-γ in the cecal tonsils (Table 12). The relative expression of these genes in the CH birds decreased in a quadratic manner (P < 0.01) with increasing SH inclusion. No significant interaction effects were observed for IL-1 in spleen, whereas INF-γ showed a linear decrease in the CH birds fed diets with increasing SH inclusions (P = 0.018). *Eimeria* challenge significantly reduced (P < 0.01) TGFβ−1 expression in the spleen and cecal tonsil (P < 0.001). A significant main effect of diet was observed on the splenic CD8:CD4 ratio, with levels decreasing linearly (P = 0.037) with increasing SH inclusion in the diet. The effect of the dietary treatments on immune response on d 28, which corresponded to the end of the recovery phase, is shown in Supplementary Table 8.

**Table 12:** Relative gene expression of pro- and anti-inflammatory cytokines in the spleen and cecal tonsils and bile immunoglobulin A content of broiler chickens (d 21) challenged or unchallenged with mixed *Eimeria* spp. and fed diets containing standard or low-protein soybean meals.

Eimeria	Diets^1^	Cecal tonsil						Spleen						Bile
		IL1	IFNɣ	IL8	LITAF	TGFβ1	CD8:CD4	IL1	IFNɣ	IL8	LITAF	TGFβ1	CD8:CD4	IgA
		Means for the main effect of *Eimeria* challenge												
Challenged		1.56	2.05	1.18	1.17	1.92	1.37	1.16	1.38	1.18	1.14	2.07	3.37	0.06
Non- challenged		1.10	1.11	1.13	1.05	1.08	1.43	1.17	1.04	1.12	1.04	1.17	4.39	0.01
		Means for the main effect of Diets												
	SSBM	2.22	2.57	1.16	0.99	1.60	1.42	1.08	1.34	1.06	1.12	1.45	4.29	0.04
	LPSBM1	0.87	0.93	0.99	1.19	1.47	1.24	1.12	1.47	1.17	1.10	1.63	3.98	0.03
	LPSBM2	1.10	1.44	1.18	1.07	1.28	1.51	1.20	1.14	1.15	0.98	1.69	3.66	0.03
	LPSBM3	1.13	1.38	1.30	1.19	1.64	1.44	1.27	0.90	1.21	1.14	1.71	3.58	0.03
		Means for the simple effects												
Challenged	SSBM	3.45	4.14	1.32	0.99	2.20	1.36	1.15	1.68	1.12	1.24	1.89	3.87	0.08
	LPSBM1	0.84	0.80	1.05	1.32	2.03	1.28	1.20	1.87	1.22	1.17	2.09	3.50	0.06
	LPSBM2	1.00	1.70	1.11	1.15	1.33	1.48	0.95	1.14	1.26	0.96	1.94	3.45	0.06
	LPSBM3	0.97	1.54	1.25	1.21	2.12	1.37	1.34	0.86	1.13	1.17	2.35	2.67	0.05
Non-challenged	SSBM	1.00	1.00	1.00	1.00	1.00	1.48	1.00	1.00	1.00	1.00	1.00	4.71	0.00
	LPSBM1	0.90	1.06	0.94	1.05	0.91	1.21	1.03	1.07	1.13	1.03	1.18	4.47	0.01
	LPSBM2	1.20	1.18	1.24	0.99	1.23	1.54	1.45	1.14	1.05	1.00	1.43	3.87	0.01
	LPSBM3	1.30	1.21	1.34	1.16	1.16	1.51	1.21	0.94	1.29	1.12	1.07	4.49	0.01
Pooled SEM		*Pooled standard error of the means*												
*Eimeria*		0.151	0.207	0.068	0.056	0.127	0.046	0.111	0.098	0.051	0.049	0.118	0.179	0.003
Diet		0.213	0.292	0.096	0.079	0.180	0.065	0.157	0.138	0.071	0.069	0.166	0.253	0.004
Interaction		0.302	0.414	0.136	0.112	0.255	0.093	0.223	0.916	0.101	0.097	0.235	0.358	0.006
		*Probabilities*												
*Eimeria*		0.036	0.003	0.598	0.142	< 0.001	0.361	0.943	0.016	0.414	0.160	< 0.001	0.000	< 0.001
Diet- Linear		0.003	0.024	0.183	0.192	0.942	0.265	0.337	0.011	0.176	0.884	0.262	0.037	0.056
Diet- Quadratic		0.002	0.010	0.139	0.630	0.181	0.423	0.918	0.189	0.703	0.186	0.631	0.658	0.262
*Eimeria* × Diet- Linear		< 0.001	0.006	0.095	0.888	0.285	0.745	0.609	0.018	0.264	0.250	0.614	0.295	0.019
*Eimeria* × Diet- Quadratic		0.008	0.009	0.506	0.216	0.202	0.323	0.327	0.729	0.252	0.463	0.259	0.214	0.108

1 Diets: SSBM- standard-protein soybean meal (464 g/kg); LPSBM - low-protein soybean meal (LPSBM1, 449 g/kg; LPSBM2,437 g/kg; LPSBM3, 417 g/kg). The LPSBM1, 2, and 3 were produced by mixing soyhull at the rates of 27, 55, or 86 g/kg, respectively, with the SSBM. IL-1, Interleukin 1; INF γ, Interferon gamma; IL-8, Interleukin-8; LITAF, Lipopolysaccharide- induced Tumor Necrosis α Factor; TGFβ−1, Transforming Growth Factor-1; CD8:CD4, cytotoxic (CD8+) to helper (CD4+) T-cells. The *Eimeria* challenge was done on d 14 with mixed species (12,500 sporulated oocysts of *E. maxima*, 12,500 sporulated oocysts of *E. tenella,* and 62,500 sporulated oocysts of *E. acervulina*) by oral gavage. n = 6 replicate pens for the simple effects, 12 replicate pens for the main effect of diets, and 24 replicate pens for the main effect of *Eimeria* challenge. Each pen had 22 birds. Linear and quadratic contrasts were used for the main effects of Diets and the *Eimeria* × Diet interaction. Data of immune response on d 28 (the end of the recovery phase) are presented in Supplementary Table 8.

## Discussion

The overriding factor in the current experiment is the increase in SH content of the diet through the incorporation of LPSBM. The three LPSBM used in the current experiment were formulated by mixing increasing levels of SH with SSBM, resulting in a progressive decrease in CP but not necessarily in total AA in the resulting SBM (designated LPSBM1-3). This approach was taken to mimic the practice of adding SH back to SBM to reduce its CP content, rather than adding SH directly to the diet, as reported in previous studies (Dilger et al., 2004; Tejeda and Kim, 2020). In addition, the SH used in this study was not heat-treated and therefore had greater trypsin inhibitor activity than the original SBM. Hence, the trypsin inhibitor activity in the LPSBM is expected to increase marginally with increasing SH inclusion.

The increased inclusion of SH in LPSBM also resulted in higher ADF and NDF in the final diet. For context, using the grower diets as an example, LPSBM1, 2, and 3 had overall SH inclusion at rates of 8.4, 17.4, and 26.8 g/kg, respectively. These inclusion levels increased ADF relative to SSBM by 14%, 29%, and 45% for LPSBM1, 2, and 3, respectively. In terms of diet formulation, greater inclusion of LPSBM than SSBM was required to maintain an isonitrogenous profile across all diets, but diets with LPSBM required lower supplemental levels of AA (Lys, Met, and Thr).

Although all the experimental diets were formulated to have similar CP and ME, increasing the dilution of SBM with SH reduced BWG and FI during the starter (d 0-10) and early grower phases (d 10-14) compared to SSBM. In contrast, previous studies have shown improvements in growth parameters during the early growth phases when SH was added directly to the diet. (Sadeghi et al., 2020) reported increased BWG and reduced FCR at 4% sunflower hull inclusion. Alternatively, Tejeda and Kim (2020) reported no change in BWG but reduced FCR at 40 g/kg SH inclusion compared to 80 g/kg. The overall dietary SH inclusion in the current experiment (at a maximum of 26.8 g/kg) was more modest than the lower level (40 g/kg) used in Tejeda and Kim (2020) study.

The findings are in agreement with the physiological role of fiber, which stimulates the production of pancreatic enzymes, releases cholecystokinin, and induces gastro-duodenal reflexes via the vagus nerve (Duke, 1992; Svihus et al., 2004). All of these contribute to nutrient digestion and absorption in the small intestine, potentially improving growth performance. In the current study, BWG and FI decreased progressively from SSBM to LPSBM 3. This reduction in BWG was observed to be mainly associated with reduced FI during the early growth phase (d 0-14), which may be attributed to the bulkiness of fiber-rich diets and the limited capacity of the immature broiler gastrointestinal tract. Furthermore, the FCR did not change among the various dietary treatments during this period. Consequently, reduced FI may have lowered the total nutrient intake, resulting in poor growth performance.

Several studies have demonstrated that insoluble fiber can positively affect the development of digestive tract function, improving nutrient digestibility and growth performance in non-challenged birds (Amerah et al., 2009; González-Alvarado et al., 2007; Jiménez-Moreno et al., 2013). However, responses under *Eimeria* challenge appear to depend on fiber source, inclusion level, and associated bioactive compounds (Noruzi et al., 2024; Sadeghi et al., 2020). The aforementioned studies did not evaluate age- and challenge-dependent effects. On the contrary, the current experiment examined the effects of dietary SH inclusion across various infection stages, which made it possible to demonstrate the phase-specific impact of the dietary treatments on growth performance. During the pre-patent phase, BWG declined linearly with increasing SH dilution of SBM, suggesting that higher fiber inclusion levels may limit nutrient availability and growth before intestinal damage occurs. The challenge effect was more evident by the end of the acute phase and showed that the infection had a stronger moderating influence on the growth performance outcome than the diet (Kasireddy et al., 2025).

During the overall phase (d 0-42), the growth performance of NCH birds was similar across all SH-fed groups, suggesting that the SH levels used in the experiment did not adversely affect overall performance. In contrast, CH birds exhibited a linear reduction in BWG and an increase in FCR at higher SH inclusion levels (i.e., LPSBM2 and 3). This response may be due to a higher content of cellulose and pectin polysaccharides in SH, which increased the dietary content of structurally complex fiber (Knudsen, 2014). At the higher inclusion rates (LPSBM 2 and 3), this complex matrix may increase the bulk of diets moving through the intestines, thereby enlarging the gastrointestinal organs (intestines, gizzard) as a part of an adaptive response, increasing the maintenance requirements, thereby diverting nutrients away from growth (Hetland et al., 2004; Nyachoti et al., 2000; Rezaei et al., 2018). This physiological outcome may play an oversized role during a disease challenge when limited nutrient intake is coupled with impaired nutrient utilization. The long-term implication of the above is demonstrated by the observation in the current experiment that, whereas the birds in the CH group that received LPSBM2 and 3 had lower overall (d 0-42) BWG and impaired FCR, there was no negative effect of SH inclusion on the overall growth performance of their NCH group counterparts.

The growth performance of broiler chickens depends, in part, on the availability of AA. At the end of the acute phase (d 21), N, Tyr, Met, Lys, and Arg digestibility were linearly decreased in CH birds fed LPSBM diets. (Lin et al., 2023) reported a reduction in ileal N digestibility and retention, irrespective of dietary supplementation. The linear reduction in AA digestibility (Lys, Met, Tyr, and Arg) observed only in challenged birds fed LPSBM diets likely indicates that the effects of *Eimeria*-induced intestinal damage are more pronounced with increased dietary fiber content (Montagne et al., 2003). The damage is precipitated when the parasites invade and proliferate within the epithelial cells of the gut, causing compromised digestion and absorption of nutrients in the small intestine, consequently increasing ileal endogenous AA losses and reducing AA digestibility (Adedokun et al., 2012). These effects could shorten the enzyme-substrate interaction and reduce nutrient availability for growth, resulting in decreased weight gain during the acute phase.

Sadeghi et al. (2020) reported increased relative gizzard weights in birds challenged with coccidiosis, along with a dietary effect where birds fed sunflower hulls or rice hulls increased the gizzard development. Similarly, in the current experiment, a challenge effect was observed, with birds in the CH group having higher relative gizzard weights, likely indicating that birds prioritize GIT development after intestinal tissue damage (Kim et al., 2017). The relative bursa weight decreased in birds fed LPSBM1, then increased at higher SH inclusion levels. Previous studies on the dietary effects of rice, soy, and sugar beet pulp in non-challenged birds showed no effect on immune tissue development, suggesting that dietary fiber may have little impact on immune tissue development (Sabour et al., 2019; Zhang et al., 2023). The concurrent increases in bursa size suggest enhanced immune activation, which may have increased nutrient demands and contributed to reduced growth performance.

The cecal SCFA profile depends upon the substrate available for microbial fermentation. In the current experiment, no significant effects of dietary treatments on SCFA concentrations were observed. However, *Eimeria* challenge significantly increased the propionate, isobutyrate, and isovalerate levels. Malabsorption and enteritis usually develop during the course of coccidiosis, leading to changes in the composition of digesta and the fermentation substrates available, along with increased levels of branched-chain fatty acids such as isobutyrate and isovalerate (Lin and Olukosi, 2021; Lin et al., 2022, 2023). The absence of dietary effects on SCFA in the present study is likely due to the infection predominating the bird's digestive capacity, coupled with the largely insoluble, poorly fermentable nature of soyhulls, which may limit their contribution to microbial fermentation and SCFA production.

The findings from the current experiment show no dietary effects on VH or CD. However, *Eimeria* challenge significantly reduced jejunal VH and increased CD, likely due to sporozoite invasion of intestinal epithelial cells, which damages the intestinal mucosa and submucosa, resulting in villi shortening, causing an overall decrease in digestion and absorption of nutrients (Perez-Carbajal et al., 2010). (Teng et al., 2020) reported similar findings, with increasing *Eimeria* challenge severity. However, LPSBM1 and LPSBM2 increased VH: CD in the NCH birds, consistent with reports in which direct dietary inclusion of SH increased the VH: CD (Zhang et al., 2023). This may be due to the increased reverse peristalsis induced by the coarse fiber, which enhances mechanical stimulation and promotes villus development, thereby improving nutrient digestibility (Sacranie et al., 2012). However, no such dietary effects were evident in CH birds, likely due to extensive epithelial damage and the inflammatory responses induced by the challenge.

The current experiment demonstrated that the *Eimeria* challenge reduced the expression of nutrient transporters (Pep T1, BO+AT, and EAAT3) and the tight junction protein occludin (OCLDN) at the peak of infection (d 21). These findings are consistent with a previous study that reported decreased expression of these nutrient transporters at the end of the acute phase of infection (Lin et al., 2022). Reduced transcript expression of nutrient transporters may partly explain the observed depression in growth performance and nutrient utilization during coccidial infection (Miska and Fetterer, 2017; Su et al., 2014). It has been proposed that the suppression of brush border membrane transporters represents a host defense strategy to limit intracellular nutrient availability and thereby “starve” the parasite, thereby limiting its replication (Su et al., 2015). The absence of effects of dietary treatment on the gene expression of tight junction and nutrient transporters suggested that, in the acute phase of the infection, *Eimeria*-induced gut damage was more pronounced than the regulatory effects of dietary fiber. Together, these alterations would impair nutrient absorption and utilization, thereby reducing AA digestibility and depressing growth performance during peak infection.

In the current experiment, dietary inclusion of SH in LPSBM1 in *Eimeria*-challenged birds significantly reduced IL-1 and IFN-γ expression during the acute phase (d21), suggesting a potential modulatory effect on excessive inflammatory signaling. In addition, LPSBM2 and LPSBM3 did not exhibit further immunomodulatory effects. Moderate SH inclusion (LPSBM1) may have reduced inflammation by decreasing antigenic exposure and enhancing immunological tolerance, as suggested by observations that SH fiber fractions alleviated TLR4/NF-κB signaling, a pathway involved in inflammation (Yu et al., 2022). Mucosal antigen stimulation may be suppressed by these mechanisms, which may also help control excessive TH1 activation. These findings indicate that relatively small dietary contributions of SH may be sufficient to influence immune responses under coccidial stress. Additionally, higher bile IgA concentrations in *Eimeria*-challenged birds on d 21 indicate the importance of mucosal antibody-mediated defenses during and after *Eimeria* infection. It has been observed that increased IgA in the bile neutralizes intestinal lumen antigens and prevents attachment by *Eimeria*, thereby preserving the intestinal mucosa over time, even after inflammation has subsided (Lillehoj and Trout, 1996). However, graded inclusion of SH in diets for *Eimeria*-challenged birds reduced IgA levels, suggesting the modulation of immune activation. Also, as antibody production requires AA, changes in IgA levels may reflect altered nutrient allocation during immune stress. Reduced digestibility of key amino acids, such as threonine, a tendency observed in the current study, may limit their availability for other immune functions, such as IgA synthesis, as they are required for mucin production during intestinal challenge and fiber inclusion (Ruth and Field, 2013). These findings suggest that increased inclusion of SH in the diet of *Eimeria*-challenged birds altered immune nutrient partitioning during the acute phase, potentially contributing to reduced performance.

The relative abundance of sulfonamide (sul2), tetracycline (tetM), streptomycin (strB), β-lactamase (blaCTX-M) resistance genes in the cecal content of broiler chickens was also evaluated in the current experiment. *Eimeria* challenge significantly increased the relative abundance of sul2 and tetM, but decreased blaCTX-M, indicating that gut infection can modulate the gut resistome. This suggests that *Eimeria* infection disrupts gut microbial populations by increasing the abundance of opportunistic bacteria, such as *E. coli* and *Clostridium* spp (Wu et al., 2014). Such shifts may favor bacterial populations harboring AMR genes, thereby contributing to an expanded gut resistome (Mak et al., 2022). However, sul2 decreased in birds fed LPSBM2 and 3, which had lower AMR than in birds fed LPSBM1. This reduced AMR gene abundance with increased dietary fiber intake may be associated with modulation of the gut microbiota, which can limit the proliferation of opportunistic bacteria that commonly harbor resistance genes.

Taken together, the findings from the current experiment demonstrate that *Eimeria* challenge significantly impaired growth performance, gut integrity, and nutrient utilization, and cecal microbial metabolites in broilers during the acute phase of infection. However, during the overall growth phase, the *Eimeria*-challenged birds fed LPSBM1 achieved performance comparable to that of birds fed the SSBM diet. The immune responses in birds receiving LPSBM1 diets were further positively modulated on d 21 by controlling excessive inflammation, potentially resulting in improved immune response outcome during the peak of infection, with moderate dietary SH inclusion. Prolonged inflammatory responses require substantial metabolic resources and shift nutrient allocation from growth to immune protein synthesis and tissue repair. Consequently, the reduction in IL-1β and IFN-γ expression in LPSBM1-CH birds reduced the inflammatory response, likely helping conserve nutrients for maintenance and recovery once the peak of infection subsided.

Hence, it can be concluded from the findings of the current experiment that moderate inclusion of soyhull into SBM, moderately increasing dietary fiber components of the protein feedstuff with soybean fiber, improves broiler growth performance and immunity under enteric stress conditions. On the other hand, within the levels of soyhull used in the current experiment, the non-challenged birds can tolerate and even benefit from the inclusion of soyhull in the diet.

## CRediT authorship contribution statement

**Bhargavi Kasireddy:** Writing – original draft, Investigation, Formal analysis, Data curation, Conceptualization. **Iyabo W. Oluseyifunmi:** Investigation. **Revathi Shanmugasundaram:** Writing – review & editing, Methodology, Investigation. **Adelumola Oladeinde:** Writing – review & editing, Methodology. **Oluyinka A. Olukosi:** Writing – review & editing, Supervision, Resources, Project administration, Methodology, Investigation, Conceptualization.

## Disclosures

The authors declare that they have no known competing financial interests or personal relationships that could have appeared to influence the work reported in this paper.

## Appendix Supplementary materials

Image, application 1

## Footnotes

Supplementary material associated with this article can be found, in the online version, at doi:10.1016/j.psj.2026.107410.

## References

[bib0001] Adedokun S., Ajuwon K., Romero L., Adeola O. (2012). Ileal endogenous amino acid losses: response of broiler chickens to fiber and mild coccidial vaccine challenge. Poult. Sci..

[bib0002] Ahsan T., Tahir M., Naz S., Khan R.U., Alhidary I.A., Abdelrahman S.H., Selvaggi M. (2024). Effect of soy hulls as alternative ingredient on growth performance, carcase quality, nutrients digestibility and intestinal histological features in broilers. Ital. J. Anim. Sci..

[bib0003] Amerah A., Ravindran V., Lentle R. (2009). Influence of insoluble fibre and whole wheat inclusion on the performance, digestive tract development and ileal microbiota profile of broiler chickens. Br. Poult. Sci..

[bib0004] Baurhoo B., Phillip L., Ruiz-Feria C. (2007). Effects of purified lignin and mannan oligosaccharides on intestinal integrity and microbial populations in the ceca and litter of broiler chickens. Poult. Sci..

[bib0005] Blake D.P., Knox J., Dehaeck B., Huntington B., Rathinam T., Ravipati V., Ayoade S., Gilbert W., Adebambo A.O., Jatau I.D. (2020). Re-calculating the cost of coccidiosis in chickens. Vet. Res..

[bib0006] Campos P.M., Miska K.B., Kahl S., Jenkins M.C., Shao J., Proszkowiec-Weglarz M. (2022). Effects of eimeria tenella on cecal luminal and mucosal microbiota in broiler chickens. Avian Dis..

[bib0007] Cobb, C.V. 2.023. 500 Broiler performance & nutrition supplement. 2022.

[bib0008] Craig A.D., Khattak F., Hastie P., Bedford M.R., Olukosi O.A. (2020). The similarity of the effect of carbohydrase or prebiotic supplementation in broilers aged 21 days, fed mixed cereal diets and challenged with coccidiosis infection. PLoS One.

[bib0009] Dasireddy J.R., Kappari L., Pender C., Doupovec B., Murugesan R., Ramirez S., Selvaraj R.K., Applegate T.J., Shanmugasundaram R. (2025). Mycotoxins and synbiotics in broilers effect of synbiotics on broilers exposed to subclinical doses of fumonisins and deoxynivalenol. Poult. Sci..

[bib0010] Dilger R., Sands J., Ragland D., Adeola O. (2004). Digestibility of nitrogen and amino acids in soybean meal with added soyhulls. J. Anim. Sci..

[bib0011] Duke G.E. (1992). Recent studies on regulation of gastric motility in turkeys. Poult. Sci..

[bib0012] Engberg R.M., Hedemann M.S., Jensen B.B. (2002). The influence of grinding and pelleting of feed on the microbial composition and activity in the digestive tract of broiler chickens. Br. Poult. Sci..

[bib0013] González-Alvarado J., Jiménez-Moreno E., Lázaro R., Mateos G. (2007). Effect of type of cereal, heat processing of the cereal, and inclusion of fiber in the diet on productive performance and digestive traits of broilers. Poult. Sci..

[bib0020] González-Alvarado J., Jiménez-Moreno E., Lázaro R., Mateos G. (2007). Effect of type of cereal, heat processing of the cereal, and inclusion of Fiber in the diet on productive performance and digestive traits of broilers. Poult. Sci..

[bib0014] Graham D., Petrone-Garcia V.M., Hernandez-Velasco X., Coles M.E., Juarez-Estrada M.A., Latorre J.D., Chai J., Shouse S., Zhao J., Forga A.J. (2023). Assessing the effects of a mixed Eimeria spp. Challenge on performance, intestinal integrity, and the gut microbiome of broiler chickens. Front. Vet. Sci..

[bib0015] Hetland H., Choct M., Svihus B. (2004). Role of insoluble non-starch polysaccharides in poultry nutrition. World's. Poult. Sci. J..

[bib0016] Hetland H., Svihus B. (2001). Effect of oat hulls on performance, gut capacity and feed passage time in broiler chickens. Br. Poult. Sci..

[bib0017] Jiménez-Moreno E., Chamorro S., Frikha M., Safaa H., Lázaro R., Mateos G.G. (2011). Effects of increasing levels of pea hulls in the diet on productive performance, development of the gastrointestinal tract, and nutrient retention of broilers from one to eighteen days of age. Anim. Feed Sci. Technol..

[bib0018] Jiménez-Moreno E., Frikha M., de Coca-Sinova A., García J., Mateos G. (2013). Oat hulls and sugar beet pulp in diets for broilers 1. Effects on growth performance and nutrient digestibility. Anim. Feed Sci. Technol..

[bib0019] Jiménez-Moreno E., González-Alvarado J., de Coca-Sinova A., Lázaro R., Mateos G. (2009). Effects of source of fibre on the development and pH of the gastrointestinal tract of broilers. Anim. Feed Sci. Technol..

[bib0021] Kasireddy B., Lourenco J., Gonzalez-Ortiz G., Bedford M.R., Olukosi O.A. (2025). Growth performance, nutrient utilization, gut integrity, short-chain fatty acids, and gene expression in Eimeria-challenged broilers receiving stimbiotics and wheat bran as an additional fiber source. Poult. Sci..

[bib0022] Kim E., Leung H., Akhtar N., Li J., Barta J., Wang Y., Yang C., Kiarie E. (2017). Growth performance and gastrointestinal responses of broiler chickens fed corn-soybean meal diet without or with exogenous epidermal growth factor upon challenge with Eimeria. Poult. Sci..

[bib0023] Knudsen K.E.B. (2014). Fiber and nonstarch polysaccharide content and variation in common crops used in broiler diets. Poult. Sci..

[bib0024] Lillehoj H.S., Trout J.M. (1996). Avian gut-associated lymphoid tissues and intestinal immune responses to Eimeria parasites. Clin. Microbiol. Rev..

[bib0025] Lin Y., Lourenco J.M., Olukosi O.A. (2023). Effects of xylanase, protease, and xylo-oligosaccharides on growth performance, nutrient utilization, short chain fatty acids, and microbiota in Eimeria-challenged broiler chickens fed high fiber diet. Anim. Nutr..

[bib0026] Lin Y., Olukosi O.A. (2021). Exogenous enzymes influenced Eimeria-induced changes in cecal fermentation profile and gene expression of nutrient transporters in broiler chickens. Animals.

[bib0027] Lin Y., Teng P.-Y., Olukosi O.A. (2022). The effects of xylo-oligosaccharides on regulating growth performance, nutrient utilization, gene expression of tight junctions, nutrient transporters, and cecal short chain fatty acids profile in Eimeria-challenged broiler chickens. Poult. Sci..

[bib0028] Livak K.J., Schmittgen T.D. (2001). Analysis of relative gene expression data using real-time quantitative PCR and the 2− ΔΔCT method. Methods.

[bib0029] Lourenco J.M., Kieran T.J., Seidel D.S., Glenn T.C., Silveira M.F.D, Callaway T.R., Stewart R.L. (2020). Comparison of the ruminal and fecal microbiotas in beef calves supplemented or not with concentrate. Plos One..

[bib0030] Mak P.H., Rehman M.A., Kiarie E.G., Topp E., Diarra M.S. (2022). Production systems and important antimicrobial resistant-pathogenic bacteria in poultry: a review. J. Anim. Sci. Biotechnol..

[bib0031] Mateos G., Jiménez-Moreno E., Serrano M., Lázaro R. (2012). Poultry response to high levels of dietary fiber sources varying in physical and chemical characteristics. J. Appl. Poult. Res..

[bib0032] Miska K., Fetterer R. (2017). The mRNA expression of amino acid and sugar transporters, aminopeptidase, as well as the di-and tri-peptide transporter PepT1 in the intestines of Eimeria infected broiler chickens. Poult. Sci..

[bib0033] Montagne L., Pluske J., Hampson D. (2003). A review of interactions between dietary fibre and the intestinal mucosa, and their consequences on digestive health in young non-ruminant animals. Anim. Feed Sci. Technol..

[bib0034] Noruzi H., Aziz-Aliabadi F., Imari Z.K. (2024). Effects of different levels of pistachio (Pistachia vera) green hull aqueous extract on performance, intestinal morphology and antioxidant capacity in Eimeria challenged broilers. Poult. Sci..

[bib0035] Nyachoti C.M., de Lange C.F., McBride B.W., Leeson S., Gabert V.M. (2000). Endogenous gut nitrogen losses in growing pigs are not caused by increased protein synthesis rates in the small intestine. J. Nutr..

[bib0036] Owusu-Asiedu A., Patience J., Laarveld B., Van Kessel A., Simmins P., Zijlstra R. (2006). Effects of guar gum and cellulose on digesta passage rate, ileal microbial populations, energy and protein digestibility, and performance of grower pigs. J. Anim. Sci..

[bib0037] Perera W., Abdollahi M., Zaefarian F., Wester T., Ravindran G., Ravindran V. (2019). Influence of inclusion level of barley in wheat-based diets and supplementation of carbohydrase on growth performance, nutrient utilisation and gut morphometry in broiler starters. Br. Poult. Sci..

[bib0038] Perez-Carbajal C., Caldwell D., Farnell M., Stringfellow K., Pohl S., Casco G., Pro-Martinez A., Ruiz-Feria C. (2010). Immune response of broiler chickens fed different levels of arginine and vitamin E to a coccidiosis vaccine and Eimeria challenge. Poult. Sci..

[bib0039] Persia M., Young E., Utterback P., Parsons C. (2006). Effects of dietary ingredients and Eimeria acervulina infection on chick performance, apparent metabolizable energy, and amino acid digestibility. Poult. Sci..

[bib0040] Rezaei M., Karimi Torshizi M., Wall H., Ivarsson E. (2018). Body growth, intestinal morphology and microflora of quail on diets supplemented with micronised wheat fibre. Br. Poult. Sci..

[bib0041] Ruth M.R., Field C.J. (2013). The immune modifying effects of amino acids on gut-associated lymphoid tissue. J. Anim. Sci. Biotechnol..

[bib0042] Sabour S., Tabeidian S.A., Sadeghi G. (2019). Dietary organic acid and fiber sources affect performance, intestinal morphology, immune responses and gut microflora in broilers. Anim. Nutr..

[bib0043] Sacranie A., Svihus B., Denstadli V., Moen B., Iji P., Choct M. (2012). The effect of insoluble fiber and intermittent feeding on gizzard development, gut motility, and performance of broiler chickens. Poult. Sci..

[bib0044] Sadeghi A., Toghyani M., Tabeidian S.A., Foroozandeh A.D., Ghalamkari G. (2020). Efficacy of dietary supplemental insoluble fibrous materials in ameliorating adverse effects of coccidial challenge in broiler chickens. Arch. Anim. Nutr..

[bib0045] Shanmugasundaram R., Kogut M.H., Arsenault R.J., Swaggerty C.L., Cole K., Reddish J.M., Selvaraj R.K. (2015). Effect of Salmonella infection on cecal tonsil regulatory T cell properties in chickens. Poult. Sci..

[bib0046] Shanmugasundaram R., Sifri M., Selvaraj R.K. (2013). Effect of yeast cell product (CitriStim) supplementation on broiler performance and intestinal immune cell parameters during an experimental coccidial infection. Poult. Sci..

[bib0047] Short F., Gorton P., Wiseman J., Boorman K. (1996). Determination of titanium dioxide added as an inert marker in chicken digestibility studies. Anim. Feed Sci. Technol..

[bib0048] Slama J., Schedle K., Wurzer G.K., Gierus M. (2019). Physicochemical properties to support fibre characterization in monogastric animal nutrition. J. Sci. Food Agric..

[bib0049] Su S., Miska K., Fetterer R., Jenkins M., Wong E. (2014). Expression of digestive enzymes and nutrient transporters in Eimeria acervulina-challenged layers and broilers. Poult. Sci..

[bib0050] Su S., Miska K., Fetterer R., Jenkins M., Wong E. (2015). Expression of digestive enzymes and nutrient transporters in Eimeria-challenged broilers. Exp. Parasitol..

[bib0051] Svihus B., Juvik E., Hetland H., Krogdahl Å. (2004). Causes for improvement in nutritive value of broiler chicken diets with whole wheat instead of ground wheat. Br. Poult. Sci..

[bib0052] Tejeda O., Kim W. (2020). The effects of cellulose and soybean hulls as sources of dietary fiber on the growth performance, organ growth, gut histomorphology, and nutrient digestibility of broiler chickens. Poult. Sci.

[bib0053] Teng P.-Y., Yadav S., de Souza Castro F.L., Tompkins Y.H., Fuller A.L., Kim W.K. (2020). Graded eimeria challenge linearly regulated growth performance, dynamic change of gastrointestinal permeability, apparent ileal digestibility, intestinal morphology, and tight junctions of broiler chickens. Poult. Sci.

[bib0054] Wu S.-B., Stanley D., Rodgers N., Swick R.A., Moore R.J. (2014). Two necrotic enteritis predisposing factors, dietary fishmeal and eimeria infection, induce large changes in the caecal microbiota of broiler chickens. Vet. Microbiol..

[bib0055] Yu C., Wang D., Yang Z., Wang T. (2022). Pharmacological effects of polyphenol phytochemicals on the intestinal inflammation via targeting TLR4/NF-κB signaling pathway. Int. J. Mol. Sci..

[bib0056] Zhang C., Hao E., Chen X., Huang C., Liu G., Chen H., Wang D., Shi L., Xuan F., Chang D. (2023). Dietary fiber level improve growth performance, nutrient digestibility, immune and intestinal morphology of broilers from day 22 to 42. Animals.

