# Supplementary material for: Growth performance and gut health of broilers fed graded inclusion levels of soyhulls in low-protein soybean meal diets and challenged with mixed Eimeria spp
Source: Poult Sci. 2026 Jul 8;105(10):107410. doi: 10.1016/j.psj.2026.107410 (PMC13400365; doi:10.1016/j.psj.2026.107410)
Supplement: Supplementary file 1 [file mmc1.docx]

Supplementary Table 1. List of primers used for real-time PCR.

| Gene symbol | Accession number | Forward primer (5′ - 3′) | Reverse primer (5′ - 3′) |
| --- | --- | --- | --- |
| GAPDH | NM_204305.1 | CCTCTCTGGCAAAGTCCAAG | GGTCACGCTCCTGGAAGATA |
| OCLN |  | ACGGCAGCACCTACCTCAA | GGCGAAGAAGCAGATGAG |
| GLUT1 |  | CTTTGTCAACCGCTTTGG | CAGAATACAGGCCG ATGAT |
| PEPT1 (SLC15A1) | NM_204365.1 | CTGGAGCATCCAAACTCA | CTTCAACCTCATTTGGATCAG |
| B^0+^AT (SLC7A9) | NM_001199133.1 | TTATCACCGCACCTGAAC | AGCATCTGAAGGTGCATAG |
| EAAT3 (SLC1A1) | XM_424930.6 | GTGATTGTTCTGAGCGCTGT | ATCCCAGTACCAAAGGCATC |
| CAT1 (SLC7A1) |  | CCAAGCACGCTGATAAAG | TACTCACAATAGGAAGAAGGG |
| IL-1β | Y15006.1 | TCCTCCAGCCAGAAAGTGA | CAGGCGGTAGAAGATGAAGC |
| IFN- γ | NM_205149.2 | CACATATCTGAGGAGCTCTATAC | GTTCATTCGCGGCTTTG |
| IL-8 |  |  |  |
| TGF β | HE646744.1 | CAGAGCATTGCCAAGAAGC | GCACGCAGCAGTTCTTCTC |
| LITAF |  |  |  |

GAPDH, glyceraldehyde-3-phosphate dehydrogenase; OCLDN, Occludin; GLUT-1, Glucose transporter-1; PEPT1, Peptide transporter 1; B0AT, Na+-dependent amino acid transporter; EAAT Excitatory amino acid transporter; CAT 1, Cationic AA transporter 1; IL-1β, Interleukin-1β; IFN- γ, Interferon γ; IL-8, Interleukin 8; TGF β, Transforming Growth Factor β;

Supplementary Table 2. List of primers for antimicrobial resistant genes

| Gene symbol | Gene name | Forward primer | Reverse primer | Reference |
| --- | --- | --- | --- | --- |
| gapA | Glyceraldehyde-3-phosphate dehydrogenase A | CCGTTGAAGTGAAAGACGGTC | AACCACTTTCTTCGCACCAGC | <https://doi.org/10.1128/mbio.02214-15> |
| tetM | Tetracycline resistance gene M | TAATATTGGAGTTTTAGCTCATGTTGATG | CCTCTCTGACGTTCTAAAAGCGTATTAT | <https://doi.org/10.1128/mbio.02214-15> |
| Sul2 | Sulphonamide resistance gene 2 | ATCGCTCATCATTTTCGG | CGAGGTCGATCACATCTG | Developed in-house |
| strB | Streptomycin resistance gene B | GCTCGGTCGTGAGAACAATCT | CAATTTCGGTCGCCTGGTAGT | <https://doi.org/10.1> |
| blaCTXM | β-lactamase CTX-M type gene | CGGGCRATGGCGCARAC | TGCRCCGGTSGTATTGCC | <https://doi.org/10.1.371/journal.pone.0100956> |

Supplementary Table 3. Analyzed crude protein and total amino acids (g/kg as fed) of the standard and low-protein soybean meals and soy hull.

| Items | SSBM^1^ | LPSBM1^1^ | LPSBM2^1^ | LPSBM3^1^ | SH^1^ |
| --- | --- | --- | --- | --- | --- |
| Crude protein | 464 | 449 | 437 | 417 | 113 |
| Indispensable amino acids |  |  |  |  |  |
| Arg | 33.7 | 33.0 | 33.0 | 32.2 | 5.4 |
| His | 12.7 | 12.4 | 12.4 | 12.2 | 3.3 |
| Ile | 22.9 | 22.5 | 22.3 | 21.8 | 4.9 |
| Leu | 36.5 | 35.9 | 35.9 | 35.0 | 7.2 |
| Lys | 30.1 | 29.7 | 29.5 | 29.0 | 8.1 |
| Met | 6.4 | 6.3 | 6.2 | 6.2 | 1.3 |
| Phe | 24.3 | 23.9 | 23.9 | 23.2 | 4.3 |
| Thr | 18.1 | 17.8 | 17.8 | 17.5 | 3.8 |
| Trp | 6.9 | 6.7 | 6.4 | 6.4 | 0.2 |
| Val | 23.4 | 22.9 | 22.8 | 22.3 | 5.1 |
| Dispensable amino acids |  |  |  |  |  |
| Ala | 20.1 | 19.7 | 19.6 | 19.3 | 4.6 |
| Asp | 53.1 | 51.9 | 52.1 | 50.9 | 10.4 |
| Cys | 6.7 | 6.5 | 6.5 | 6.5 | 2.0 |
| Glu | 85.7 | 83.7 | 83.6 | 81.9 | 12.1 |
| Gly | 19.7 | 19.3 | 19.4 | 19.1 | 8.8 |
| Prol | 24.0 | 24.1 | 23.6 | 23.2 | 7.5 |
| Seri | 20.9 | 20.5 | 20.5 | 20.2 | 5.5 |
| Tyr | 17.0 | 17.0 | 16.7 | 16.3 | 5.0 |
| DM | 951 | 890 | 880 | 890 | 956 |
| NDF^2^ | 15.4 | 30.39 | 45.33 | 60.32 | 586 |
| ADF^2^ | 16.3 | 27.25 | 38.17 | 49.13 | 433 |
| Cellulose |  |  |  |  | 422 |
| Hemicellulose |  |  |  |  | 153 |
| Raffinose | 13.5 |  |  |  |  |
| Stachyose | 74.4 |  |  |  |  |
| Verbascose | 2.4 |  |  |  |  |
| TIU^3^ | 15.4 |  |  |  | 116 |

^1^Diets: SSBM- standard-protein soybean meal (464 g/kg); LPSBM - low-protein soybean meal (LPSBM1, 449 g/kg; LPSBM2,437 g/kg; LPSBM3, 417 g/kg). The LPSBM1, 2, and 3 were produced by mixing soyhull at the rates of 27, 55, or 86 g/kg, respectively, with the SSBM

TIU - trypsin inhibitor unit (expressed per mg protein)

Supplementary Table 4. Growth performance response (interaction effects) of broiler chickens challenged or unchallenged with mixed *Eimeria* spp. and fed diets with low-protein soybean meal during pre-patent, acute, and recovery phases of infection.

|  |  | Pre-patent phase (d14-17) | | | | Acute phase (d17-21) | | | | Recovery phase (d21-28) | | | |
| --- | --- | --- | --- | --- | --- | --- | --- | --- | --- | --- | --- | --- | --- |
|  | Treatments^1^ | Weight gain, g | Feed intake, g | FCR | Day 18 BW, g | Weight gain, g | Feed intake, g | FCR | Day 21 BW, g | Weight gain, g | Feed intake, g | FCR | Day 28 BW, g |
| Challenged | SSBM | 314 | 425 | 1.36 | 854 | 50 | 220 | 4.77 | 898 | 699 | 936 | 1.34 | 1596 |
|  | LPSBM1 | 306 | 428 | 1.40 | 807 | 50 | 207 | 8.24 | 855 | 698 | 994 | 1.42 | 1551 |
|  | LPSBM2 | 284 | 401 | 1.41 | 758 | 49 | 208 | 4.36 | 807 | 665 | 1180 | 1.78 | 1471 |
|  | LPSBM3 | 284 | 409 | 1.45 | 755 | 50 | 194 | 5.13 | 805 | 648 | 1043 | 1.62 | 1451 |
| Non-challenged | SSBM | 308 | 420 | 1.37 | 809 | 288 | 405 | 1.41 | 1097 | 764 | 1249 | 1.64 | 1851 |
|  | LPSBM1 | 301 | 419 | 1.39 | 800 | 303 | 402 | 1.33 | 1103 | 713 | 1084 | 1.52 | 1806 |
|  | LPSBM2 | 298 | 407 | 1.37 | 771 | 292 | 402 | 1.38 | 1062 | 740 | 1214 | 1.63 | 1792 |
|  | LPSBM3 | 285 | 401 | 1.41 | 758 | 287 | 397 | 1.39 | 1044 | 731 | 1049 | 1.44 | 1758 |
|  | Pooled SEM | 7.21 | 7.90 | 0.025 | 18.1 | 10.8 | 10.1 | 1.624 | 23.4 | 14.9 | 97.0 | 0.134 | 28.1 |
|  |  | Probabilities | | | | | | | | | | | |
| *Eimeria* × Diet- Linear | | 0.371 | 0.889 | 0.248 | 0.153 | 0.817 | 0.391 | 0.787 | 0.390 | 0.233 | 0.118 | 0.055 | 0.213 |
| *Eimeria* × Diet- Quadratic | | 0.473 | 0.638 | 0.699 | 0.359 | 0.491 | 0.974 | 0.548 | 0.336 | 0.178 | 0.484 | 0.649 | 0.870 |

^1^Diets: SSBM- standard-protein soybean meal (464 g/kg); LPSBM - low-protein soybean meal (LPSBM1, 449 g/kg; LPSBM2,437 g/kg; LPSBM3, 417 g/kg). The LPSBM1, 2, and 3 were produced by mixing soyhull at the rates of 27, 55, or 86 g/kg, respectively, with the SSBM.

The *Eimeria* challenge was done on d 14 with mixed species (12,500 sporulated oocysts of *E. maxima*, 12,500 sporulated oocysts of *E. tenella,* and 62,500 sporulated oocysts of *E. acervulina*) by oral gavage.

n = 6 replicate pens per treatment, and each pen had 22 birds per replicate.

Linear and quadratic contrasts were used to evaluate the treatment responses.

Supplementary Table 5. Ileal dry matter, nitrogen, and amino acid digestibility for broiler chickens challenged or unchallenged with mixed *Eimeria* spp. and fed diets with a low-protein soybean meal on day 14.

|  | Dispensable amino acid digestibility | | | | | | | | | |
| --- | --- | --- | --- | --- | --- | --- | --- | --- | --- | --- |
| Diets^1^ | DMD | Asp | Ser | Glu | Pro | Gly | Ala | Cys | Tyr |  |
| SSBM | 69.1 | 81.1 | 79.6 | 86.2 | 77.9 | 75.5 | 79.4 | 66.4 | 82.4 |  |
| LPSBM1 | 69.3 | 80.4 | 79.5 | 86.1 | 77.6 | 75.0 | 80.0 | 63.1 | 81.2 |  |
| LPSBM2 | 71.4 | 82.6 | 80.9 | 87.6 | 80.0 | 77.0 | 81.4 | 68.6 | 83.8 |  |
| LPSBM3 | 68.8 | 81.0 | 78.0 | 86.9 | 78.4 | 74.9 | 80.3 | 64.0 | 81.3 |  |
| Pooled SEM | 1.817 | 1.131 | 1.301 | 0.812 | 1.296 | 1.524 | 1.346 | 2.309 | 1.058 |  |
|  | Probabilities | | | | | | | | |  |
| SSBM vs LPSBM | 0.753 | 0.870 | 0.979 | 0.489 | 0.592 | 0.967 | 0.454 | 0.648 | 0.832 |  |
| Diet- Linear | 0.922 | 0.715 | 0.627 | 0.349 | 0.516 | 0.989 | 0.510 | 0.866 | 0.924 |  |
| Diet- Quadratic | 0.460 | 0.730 | 0.297 | 0.739 | 0.604 | 0.605 | 0.537 | 0.758 | 0.568 |  |
|  | Indispensable amino acid digestibility | | | | | | | | | |
| Treatment^1^ | Thr | Val | Met | Ile | Leu | Phe | Lys | His | Arg | Trp |
| SSBM | 77.0 | 79.6 | 89.3 | 80.8 | 80.5 | 82.1 | 86.6 | 81.5 | 89.1 | 82.5 |
| LPSBM1 | 74.3 | 80.1 | 89.1 | 80.8 | 80.8 | 82.0 | 85.8 | 81.5 | 88.6 | 82.0 |
| LPSBM2 | 76.5 | 81.4 | 89.9 | 82.4 | 82.5 | 83.9 | 87.0 | 82.8 | 89.8 | 84.6 |
| LPSBM3 | 72.7 | 80.8 | 89.5 | 82.1 | 81.8 | 82.8 | 85.6 | 81.2 | 88.8 | 82.0 |
| Pooled SEM | 1.562 | 1.279 | 0.765 | 1.302 | 1.342 | 1.139 | 0.909 | 1.197 | 0.565 | 1.098 |
|  | Probabilities | | | | | | | | | |
| SSBM vs LPSBM | 0.190 | 0.462 | 0.830 | 0.486 | 0.403 | 0.518 | 0.643 | 0.834 | 0.954 | 0.731 |
| Diet- Linear | 0.160 | 0.444 | 0.683 | 0.328 | 0.338 | 0.439 | 0.649 | 0.963 | 0.916 | 0.795 |
| Diet- Quadratic | 0.724 | 0.686 | 0.925 | 0.897 | 0.675 | 0.619 | 0.730 | 0.506 | 0.681 | 0.317 |

^1^Diets: SSBM- standard-protein soybean meal (464 g/kg); LPSBM - low-protein soybean meal (LPSBM1, 449 g/kg; LPSBM2,437 g/kg; LPSBM3, 417 g/kg). The LPSBM1, 2, and 3 were produced by mixing soyhull at the rates of 27, 55, or 86 g/kg, respectively, with the SSBM.

The *Eimeria* challenge was done on d 14 with mixed species (12,500 sporulated oocysts of *E. maxima*, 12,500 sporulated oocysts of *E. tenella,* and 62,500 sporulated oocysts of *E. acervulina*) by oral gavage.

n = 6 replicate pens per treatment, and each pen had 22 birds per replicate.

Linear and quadratic contrasts were used to evaluate the treatment responses.

Supplementary Table 6. Short-chain fatty acid profile (interaction effects) of broiler chickens challenged or unchallenged with mixed *Eimeria* spp. and fed diets with a low-protein soybean meal on day 21.

| *Eimeria* | Diets^1^ | Acetate | Propionate | Isobutyrate | Butyrate | Isovalerate | Valerate | Total SCFA |
| --- | --- | --- | --- | --- | --- | --- | --- | --- |
| Challenged | SSBM | 79.7 | 8.9 | 1.5 | 20.9 | 2.1 | 1.8 | 115 |
|  | LPSBM1 | 81.6 | 9.1 | 1.8 | 15.5 | 2.4 | 2.0 | 112 |
|  | LPSBM2 | 78.3 | 9.0 | 1.5 | 20.7 | 2.4 | 1.6 | 114 |
|  | LPSBM3 | 77.5 | 9.2 | 1.5 | 17.3 | 2.2 | 1.7 | 109 |
| Non- challenged | SSBM | 86.9 | 4.1 | 0.6 | 23.1 | 0.6 | 1.1 | 116 |
|  | LPSBM1 | 74.8 | 4.1 | 0.8 | 17.2 | 0.8 | 1.0 | 98.6 |
|  | LPSBM2 | 72.7 | 3.5 | 0.7 | 17.6 | 0.7 | 1.0 | 96.3 |
|  | LPSBM3 | 74.3 | 3.9 | 0.8 | 15.8 | 0.8 | 1.0 | 96.5 |
|  | Pooled SEM | 5.536 | 1.118 | 0.168 | 3.490 | 0.358 | 0.210 | 10.25 |
|  | P-values | 0.594 | <0.001 | <0.001 | 0.674 | <0.001 | <0.001 | 0.310 |
|  | *Eimeria* × Diet- Linear | 0.407 | 0.777 | 0.571 | 0.391 | 0.926 | 0.765 | 0.390 |
|  | *Eimeria* × Diet- Quadratic | 0.323 | 0.902 | 0.532 | 0.813 | 0.648 | 0.786 | 0.429 |

^1^Diets: SSBM- standard-protein soybean meal (464 g/kg); LPSBM - low-protein soybean meal (LPSBM1, 449 g/kg; LPSBM2,437 g/kg; LPSBM3, 417 g/kg). The LPSBM1, 2, and 3 were produced by mixing soyhull at the rates of 27, 55, or 86 g/kg, respectively, with the SSBM

The *Eimeria* challenge was done on d 14 with mixed species (12,500 sporulated oocysts of *E. maxima*, 12,500 sporulated oocysts of *E. tenella,* and 62,500 sporulated oocysts of *E. acervulina*) by oral gavage.

n = 6 replicate pens per treatment, and each pen had 22 birds per replicate.

Linear and quadratic contrasts were used to evaluate the treatment responses.

Supplementary Table 7. Jejunal tight junction proteins, nutrient transporters, and antimicrobial-resistant genes of broiler chickens challenged or unchallenged with mixed *Eimeria* spp. and fed diets with a low-protein soybean meal on day 21.

| *Eimeria* | Diets^1^ | Nutrient transporter genes | | | | | Tight junction  proteins | Anti-microbial resistant genes | | | |
| --- | --- | --- | --- | --- | --- | --- | --- | --- | --- | --- | --- |
|  |  | Pep T1^2^ | CAT1^2^ | BO+AT^2^ | EAAT3^2^ | GLUT1^2^ | OCLDN^2^ | sul2^2^ | tetM^2^ | blaCTXM^2^ | strB^2^ |
| Challenged | SSBM | 0.41 | 0.50 | 0.36 | 0.48 | 2.05 | 0.47 | 0.88 | -0.67 | -0.41 | 1.25 |
|  | LPSBM1 | 0.60 | 0.64 | 0.53 | 0.51 | 2.48 | 0.48 | 1.02 | -0.98 | 0.02 | 1.09 |
|  | LPSBM2 | 0.50 | 0.84 | 0.34 | 0.53 | 2.74 | 0.54 | 0.75 | -0.88 | -0.54 | 0.61 |
|  | LPSBM3 | 0.49 | 0.58 | 0.43 | 0.51 | 2.41 | 0.49 | 0.71 | -1.28 | -0.94 | 1.03 |
| Non-challenged | SSBM | 1.00 | 1.00 | 0.75 | 1.00 | 1.00 | 0.88 | -0.83 | -2.16 | 0.76 | -0.02 |
|  | LPSBM1 | 0.90 | 0.87 | 0.74 | 0.87 | 0.91 | 0.81 | 0.91 | -1.46 | 0.88 | 1.48 |
|  | LPSBM2 | 1.32 | 0.67 | 1.03 | 1.05 | 0.64 | 0.98 | 0.51 | -3.22 | 0.76 | 0.38 |
|  | LPSBM3 | 1.24 | 0.68 | 1.13 | 1.08 | 1.34 | 0.95 | -0.85 | -2.62 | 0.21 | 0.09 |
|  | Pooled SEM | 0.189 | 0.138 | 0.166 | 0.196 | 0.496 | 0.112 | 0.566 | 0.927 | 0.487 | 0.477 |
|  | P-values |  |  |  |  |  |  |  |  |  |  |
|  | *Eimeria* × Diet- Linear | 0.370 | 0.060 | 0.156 | 0.777 | 0.812 | 0.702 | 0.925 | 0.814 | 0.900 | 0.901 |
|  | *Eimeria* × Diet- Quadratic | 0.667 | 0.157 | 0.663 | 0.655 | 0.214 | 0.746 | 0.076 | 0.997 | 0.911 | 0.087 |

^1^Diets: SSBM- standard-protein soybean meal (464 g/kg); LPSBM - low-protein soybean meal (LPSBM1, 449 g/kg; LPSBM2,437 g/kg; LPSBM3, 417 g/kg). The LPSBM1, 2, and 3 were produced by mixing soyhull at the rates of 27, 55, or 86 g/kg, respectively, with the SSBM.

^2^Pep t1, Peptide transporter 1; CAT 1, Cationic amino acid transporter 1; BO+AT, Neutral amino acid transporter; EAAT3, Excitatory amino acid transporter; GLUT 1, Glucose transporter 1; OCLDN, Occludin; sul2- Sulphonamide resistance gene 2; tetM-Tetracycline resistance gene M; blaCTX-M- β-lactamase CTX-M type gene; strB -Streptomycin resistance gene B, gapA- glyceraldehyde-3-phosphate dehydrogenase.

The *Eimeria* challenge was done on d 14 with mixed species (12,500 sporulated oocysts of *E. maxima*, 12,500 sporulated oocysts of *E. tenella,* and 62,500 sporulated oocysts of *E. acervulina*) by oral gavage.

n = 6 replicate pens per treatment, and each pen had 22 birds per replicate.

Linear and quadratic contrasts were used to evaluate treatment responses.

Supplementary Table 8. Pro- and anti-inflammatory cytokine gene expression of spleen and cecal tonsils in broiler chickens challenged or unchallenged with mixed *Eimeria* spp. and fed diets with a low-protein soybean meal on day 28.

| *Eimeria* | Diets |  | D 28 cecal tonsil | | | | | | D 28 Spleen | | | | |  |
| --- | --- | --- | --- | --- | --- | --- | --- | --- | --- | --- | --- | --- | --- | --- |
|  |  | IL1 | IFNg | IL8 | LITAF | TGFB | CD8:  CD4 | IL1 | IFNg | IL8 | LITAF | TGFB | CD8:  CD4 | Bile IgA |
|  |  | Means for the main effect of Eimeria challenge | | | | | | | | | | | | |
| Challenged | | 1.08 | 1.24 | 1.06 | 1.21 | 1.57 | 1.23 | 1.09 | 1.07 | 1.11 | 1.12 | 1.00 | 2.45 | 0.06 |
| Non- challenged | | 1.01 | 0.76 | 1.03 | 1.04 | 1.11 | 1.22 | 0.96 | 1.05 | 1.08 | 1.04 | 1.02 | 3.66 | 0.01 |
|  |  | Means for the main effect of Diets | | | | | | | | | | | | |
|  | SSBM | 0.99 | 1.95 | 1.02 | 1.10 | 1.93 | 1.24 | 1.02 | 1.03 | 1.08 | 1.03 | 1.08 | 2.71 | 0.03 |
|  | LPSBM1 | 1.13 | 0.66 | 1.13 | 1.18 | 1.17 | 1.21 | 0.98 | 0.85 | 1.09 | 1.16 | 0.77 | 2.43 | 0.03 |
|  | LPSBM2 | 1.03 | 0.81 | 1.03 | 1.16 | 1.18 | 1.22 | 1.06 | 1.24 | 1.14 | 1.05 | 1.07 | 3.26 | 0.04 |
|  | LPSBM3 | 1.04 | 0.58 | 1.00 | 1.06 | 1.08 | 1.22 | 1.02 | 1.11 | 1.07 | 1.08 | 1.13 | 3.82 | 0.04 |
|  |  | Means for the simple effects | | | | | | | | | | | | |
| Challenged | SSBM | 0.97 | 2.91 | 1.03 | 1.20 | 2.85 | 1.19 | 1.04 | 1.06 | 1.15 | 1.07 | 1.15 | 2.60 | 0.06 |
|  | LPSBM1 | 1.41 | 0.68 | 1.16 | 1.41 | 1.31 | 1.23 | 1.00 | 0.76 | 1.17 | 1.34 | 0.59 | 2.11 | 0.06 |
|  | LPSBM2 | 0.93 | 0.84 | 1.12 | 1.27 | 1.23 | 1.27 | 1.16 | 1.42 | 1.13 | 1.07 | 1.18 | 2.79 | 0.06 |
|  | LPSBM3 | 1.00 | 0.54 | 0.93 | 0.96 | 0.89 | 1.24 | 1.15 | 1.04 | 0.98 | 1.00 | 1.10 | 2.28 | 0.07 |
| Non-challenged | SSBM | 1.00 | 1.00 | 1.00 | 1.00 | 1.00 | 1.29 | 1.00 | 1.00 | 1.00 | 1.00 | 1.00 | 2.81 | 0.01 |
|  | LPSBM1 | 0.85 | 0.64 | 1.10 | 0.95 | 1.03 | 1.20 | 0.96 | 0.93 | 1.01 | 0.99 | 0.96 | 2.74 | 0.01 |
|  | LPSBM2 | 1.13 | 0.78 | 0.94 | 1.05 | 1.13 | 1.18 | 0.97 | 1.06 | 1.15 | 1.03 | 0.97 | 3.72 | 0.01 |
|  | LPSBM3 | 1.08 | 0.63 | 1.06 | 1.16 | 1.27 | 1.20 | 0.90 | 1.19 | 1.16 | 1.15 | 1.17 | 5.36 | 0.02 |
|  |  | *Pooled SEM* | | | | | | | | | | | | |
| *Eimeria* |  | 0.070 | 0.130 | 0.054 | 0.071 | 0.091 | 0.022 | 0.078 | 0.072 | 0.071 | 0.072 | 0.073 | 0.498 | 0.002 |
| Diets |  | 0.098 | 0.183 | 0.077 | 0.101 | 0.130 | 0.031 | 0.110 | 0.103 | 0.100 | 0.101 | 0.103 | 0.705 | 0.003 |
| Interaction |  | 0.139 | 0.260 | 0.108 | 0.143 | 0.184 | 0.044 | 0.156 | 0.143 | 0.142 | 0.143 | 0.145 | 0.997 | 0.004 |
|  |  | *Probabilities* | | | | | | | | | | | | |
| *Eimeria* | | 0.501 | 0.012 | 0.677 | 0.101 | 0.001 | 0.611 | 0.258 | 0.825 | 0.779 | 0.462 | 0.853 | 0.093 | <0.001 |
| Diet- Linear | | 0.892 | <0.001 | 0.634 | 0.763 | <0.001 | 0.649 | 0.848 | 0.167 | 0.956 | 0.971 | 0.307 | 0.192 | 0.135 |
| Diet- Quadratic | | 0.496 | 0.006 | 0.325 | 0.373 | 0.015 | 0.668 | 0.994 | 0.775 | 0.662 | 0.618 | 0.085 | 0.553 | 0.263 |
| *Eimeria* × Diet- Linear | | 0.314 | 0.001 | 0.557 | 0.376 | <0.001 | 0.111 | 0.434 | 0.884 | 0.193 | 0.301 | 0.923 | 0.165 | 0.837 |
| *Eimeria* × Diet- Quadratic | | 0.242 | 0.025 | 0.271 | 0.103 | 0.044 | 0.160 | 0.881 | 0.483 | 0.668 | 0.252 | 0.573 | 0.544 | 0.601 |

^1^Diets: SSBM- standard-protein soybean meal (464 g/kg); LPSBM - low-protein soybean meal (LPSBM1, 449 g/kg; LPSBM2,437 g/kg; LPSBM3, 417 g/kg). The LPSBM1, 2, and 3 were produced by mixing soyhull at the rates of 27, 55, or 86 g/kg, respectively, with the SSBM.

^2^IL-1, Interleukin 1; INF γ, Interferon gamma; IL-8, Interleukin-8; LITAF, Lipopolysaccharide- induced Tumor Necrosis α Factor; TGFβ-1, Transforming Growth Factor-1; CD8:CD4, cytotoxic (CD8+) to helper (CD4+) T-cells.

The *Eimeria* challenge was done on d 14 with mixed species (12,500 sporulated oocysts of *E. maxima*, 12,500 sporulated oocysts of *E. tenella,* and 62,500 sporulated oocysts of *E. acervulina*) by oral gavage.

n = 6 replicate pens per treatment, and each pen had 22 birds per replicate.

Linear and quadratic contrasts were used to evaluate treatment responses.
